# Evaluation of tumor response to immune checkpoint inhibitors by a 3D immunotumoroid model

**DOI:** 10.3389/fimmu.2024.1356144

**Published:** 2024-03-28

**Authors:** Abdulmohammad Pezeshki, John C. Cheville, Angela B. Florio, Bradley C. Leibovich, George Vasmatzis

**Affiliations:** ^1^ Biomarker Discovery, Mayo Clinic, Rochester, MN, United States; ^2^ Department of Molecular Medicine, Mayo Clinic, Rochester, MN, United States; ^3^ Department of Laboratory Medicine and Pathology, Mayo Clinic, Rochester, MN, United States; ^4^ Urology, Mayo Clinic, Rochester, MN, United States

**Keywords:** immune checkpoint inhibitor (ICI), immunotumoroid, prediction of response to immune checkpoint inhibitor (ICI) therapy, tumor-infiltrating lymphocytes (TILs), microcancer, patient derived tumor spheroid

## Abstract

**Background:**

Only 20 percent of renal and bladder cancer patients will show a significant response to immune checkpoint inhibitor (ICI) therapy, and no test currently available accurately predicts ICI response.

**Methods:**

We developed an “immunotumoroid” cell model system that recapitulates the tumor, its microenvironment, and necessary immune system components in patient-derived spheroids to enable ex vivo assessment of tumor response to ICI therapy. Immunotumoroids were developed from surgically resected renal cell carcinomas and bladder carcinomas selected for high tumor-infiltrating lymphocytes (TILs) and survived more than a month without media exchange. Immunohistochemistry was used to detect immune and non-immune cells in cryopreserved source tumors and the resulting immunotumoroids. Immunotumoroid response to ICIs (nivolumab, pembrolizumab, and durvalumab) and chemotherapy (cisplatin, gemcitabine, and paclitaxel) was monitored in real-time with Cytotox Red staining in an Incucyte device, and the immunotumoroid response was compared to retrospective clinical drug responses.

**Results:**

Six of the 13 cases tested grew viable immunotumoroid models, with failed cases attributed to extensive tumor tissue necrosis or excess lymphocytes preventing spheroid formation. One successfully cultured case was excluded from the study due to low TIL infiltration (<5%) in the primary tumor sample. The five remaining models contained immune cells (CD4+ and CD8+ T cells, and macrophages), non-immune cells (fibroblasts), and tumor cells. Chemotherapy and ICI drugs were tested in immunotumoroids from 5 cases and compared to clinical outcomes where data was available. Four/five models showed cell killing in response to chemotherapy and two/five showed sensitivity to ICI. In three cases, the immunotumoroid model accurately predicted the patient’s clinical response or non-response to ICIs or chemotherapy.

**Conclusion:**

Our immunotumoroid model replicated the multicellular nature of the tumor microenvironment sufficiently for preclinical ICI screening. This model could enable valuable insights into the complex interactions between cancer cells, the immune system, and the microenvironment. This is a feasibility study on a small number of cases, and additional studies with larger case numbers are required including correlation with clinical response.

## Introduction

Patients with bladder and renal cancers may be treated with surgical resection, chemotherapy radiation therapy, or a combination of these treatments. However, in recent years, immune checkpoint inhibitor (ICI) therapy has emerged as a promising new treatment option including in the neoadjuvant setting for bladder cancer. Immune checkpoint proteins are localized to the surface of immune cells and act as “brakes” to prevent autoimmune responses. Tumor cells often produce ligands to these checkpoints as a mechanism of immune evasion; by blocking the immune checkpoints and preventing tumor cells from interacting with them, immunotherapy can help restore the immune system’s ability to recognize and attack cancer cells. Preclinical evaluation of the tumoral response to ICI therapy can provide valuable insights into the complex interactions between cancer cells, the immune system, and the tumor microenvironment (TME).

Microcancer models (1) are 3D tumor spheroid cultures that can be used to evaluate how a tumor responds to various treatments. Spheroids recapitulate *in vivo* tumor structure including the presence of an inner hypoxic region that is characteristic of solid tumors, allowing for a physiologically relevant representation of the tumor microenvironment compared to traditional 2D cell culture systems (2, 3). The spheroids can be placed in 96 well plates and immune checkpoint inhibitors can be added to the wells, and their effects on tumor growth, immune cell cytotoxicity, and the overall response of the cancer cells can be measured. Conventionally, microcancers used for drug screening are prepared from a tumor cell line instead of the patient’s specific tumor cells (3). These tumor spheroids are usually monocellular, with effector immune cells added after aggregation to allow evaluation of the immune response (4–6).

Here we present an immunotumoroid model designed for the evaluation of ICI therapies on primary tumor cells acquired by biopsy or surgical resection. After tissue dissociation, tumor cells, stromal cells, and tumor-infiltrating lymphocytes (TILs) reassemble into a spheroid microcancer in a hanging drop culture. This model is scaffold‐free in that cells produce their own extracellular matrix (ECM) and there is no exogenous artificial ECM that could affect the drug response (2). Our immunotumoroid model reflects the *in vivo* tumor heterogeneity of tumor cells, immune cells, and ECM and allows scalability of testing with standardization of measurements.

## Methods

### Tumor collection and cryopreservation

Surgically resected tumor samples (kidney and bladder cancer) were retrieved from the Mayo Clinic operating rooms by dedicated laboratory assistants immediately upon removal from the patient. The sample was rapidly processed for clinical care and tumor in excess of diagnosis was collected for this study. Tumor samples were cryopreserved by dicing into 1 mm cubes placing them in 1.5mL cryovials and suspending them in 1.0 mL CryoStor media (07955; Stemcell) per tube. The vials were placed in freezing containers (5100-0001; Thermo Fisher Scientific), and cooled the cells at a rate of -1°C/min until reaching -80°C. After overnight incubation at -80°C, the cryopreserved tumors were stored in liquid nitrogen for later use.

### Immunotumoroid development

The cryopreserved tumors were mechanically and enzymatically dissociated using the Miltenyi Biotec tumor dissociation kit (#130-095-929) and the GentleMACS ™ Dissociator (#130-093-235) based on the manufacturer’s protocol. To generate the multicellular microcancer (immunotumoroid), 1.5 x 10 ^4^ cells/well were loaded on a 96-well GravityPLUS Hanging Drop plate (InSphero, Schlieren, Switzerland) and incubated at 37°C. Cells were seeded at 1.5 x 10 ^4^ cells/well to increase the inclusion of non-tumor cells, such as stromal cells and tumor-infiltrating lymphocytes. After 4 days of incubation (day -4), the aggregated microcancers were dropped into a 96-Well clear round bottom ultra-low attachment (ULA) plate (#7007 Corning) and cultured in DMEM/F12 media supplemented with 10% heat-inactivated human AB sera, primocin, glutamax, hepes, N-Acetyl-L-cysteine, Nicotinamide, B-27 Supplement, A83-01, insulin, EGF, and the therapeutic agents. The total volume of media per well was 240 μL. Cultures were incubated in a humidified atmosphere of 5% CO2 at 37°C.

### Real-time monitoring of drug response in the immunotumoroid model

Initial cell viability was measured on day 0, after dropping the immunotumoroid but before drug dosing, and then evaluated at later time points by Incucyte Cytotox Red staining (#4632 Sartorius). Incucyte Cytotox Red is a highly sensitive nucleic acid dye for real-time quantification of cell death. As cell health begins to decline, the integrity of the plasma membrane is compromised, allowing the dye to enter the cell. Fluorescence increases 100-1000-fold upon binding to DNA. For each drug, we applied 5 log concentrations and just the C_max_ concentration indicated in the main figures of the paper. The response to the other drug concentrations can be found in the **Supplementary Figures**. The C_max_ of the drugs was obtained from relevant publications (7) and also the Micromedex website (https://www.micromedexsolutions.com/). The microcancer response to treatment was recorded by the Incucyte S3 Live-Cell Analysis Instrument (Sartorius, Germany) using the Incucyte Spheroid Analysis Software Module (# 9600-0019).

Cisplatin (Fresenius Kabi USA, LLC), gemcitabine (Athenex Pharmaceutical Division, LLC), and paclitaxel (Athenex Pharmaceutical Division, LLC) were used as chemotherapy drugs. For ICI drugs, pembrolizumab (Merck Sharp & Dohme LLC), nivolumab (E.R. Squibb & Sons, LLC), and durvalumab (AstraZeneca Pharmaceuticals LP) were used.

### Immunohistochemistry

Immunohistochemical staining of 5 μm thick paraffin-embedded tissue sections was performed on an automated BenchMark ULTRA IHC/ISH Staining Module (Ventana Medical Systems, AZ) as follows. CD3 clone LN10 at 1/250 dilution, pretreatment with CC1 (Catalog # 950-224) 32 min at 100C, antibody for 32min at 36C, Optiview DAB detection (Catalog # 760-700). CD4 clone SP35, predilute, pretreatment with CC1 36 min at 95C, antibody for 32min at 36C, Ultraview DAB detection (Catalog # of 760-500). CD8 clone C8/144B at 1/250 dilution, pretreatment with CC1 32 min at 100C, antibody for 16 min at 36C, Optiview DAB detection. CD68 clone PG-M1 at 1/75 dilution with CC1 36 min at 95C, antibody for 32 min at 36C, Ultraview DAB detection. Vimentin clone V9, prediluted, pretreatment with CC1 32 min at 100C, antibody for 16 min at 36C, Optiview DAB detection. PD-L1 clone 22C3 at 1/50 dilution, pretreatment with CC1 64 min at 100C, antibody for 24min at 36C, Optiview +Amp DAB detection (Catalog # 760-099). PD-1 clone NAT105 at 1/300 dilution, pretreatment with CC1 32 min at 100C, antibody for 32 min at 36C, Optiview +Amp DAB detection.

### Statistical analysis

Statistical analyses were performed using GraphPad Prism. The unpaired *t* test was used to determine the statistical significance between the two groups.

## Results

### The immunotumoroid model is optimized for the extended culture time required to monitor ICI response

**Figure 1:**
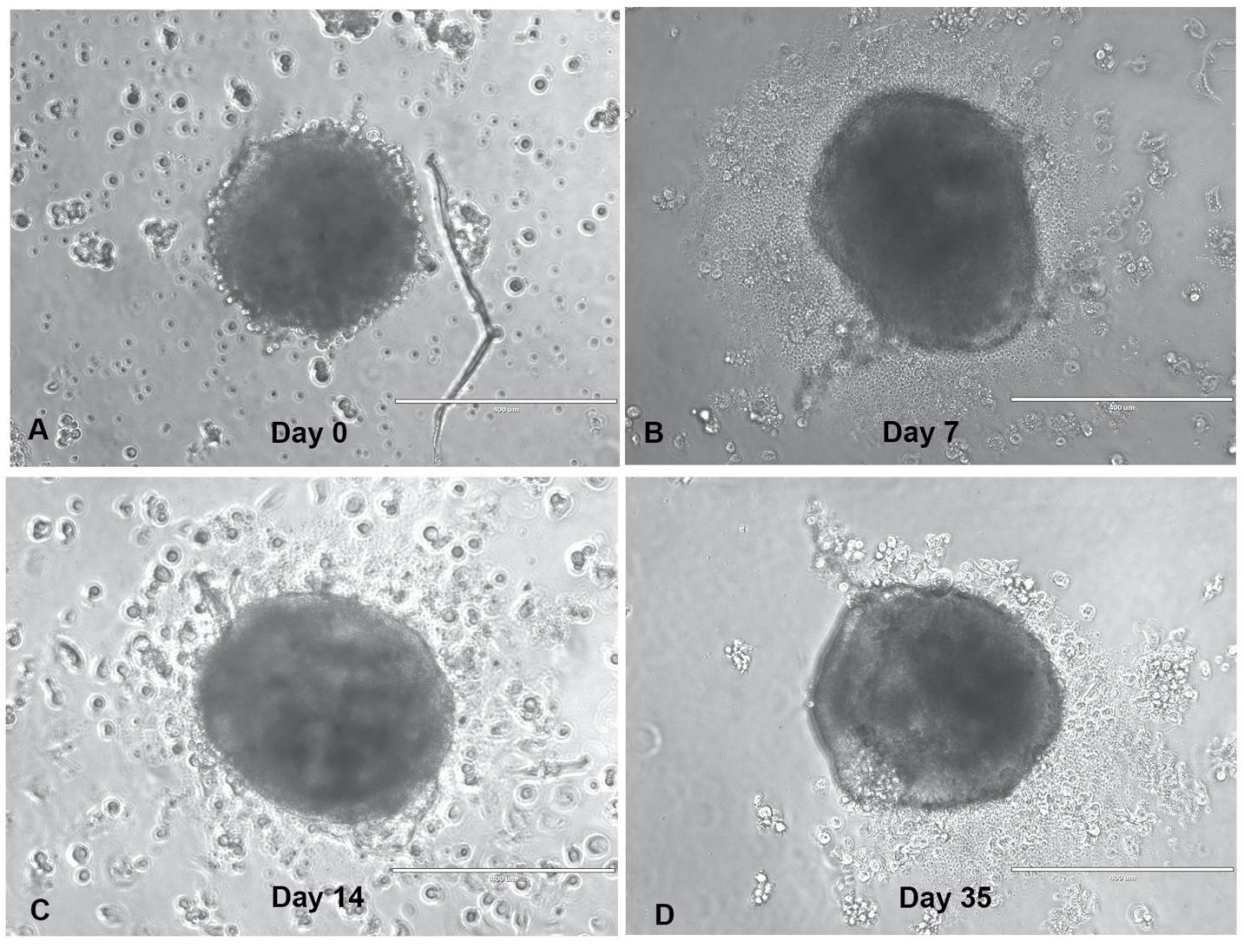
The immunotumoroid model survives long-term culture. The dissociated tumor cells were seeded as hanging drop spheroids for 4 days (day -4), and then dropped into a ULA plate in a total volume of 240 μL. The drop day was marked as day 0-time point **(A)** for drug screening purposes; panels **(B–D)** depict the immunotumoroid 7, 14, and 35 days later. Scale bar 400 μm.

This study evaluated the anti-tumor immune response of TILs, recognizing that the immunotumoroid model lacks a complete immune system and that the TILs’ anti-tumor cytotoxicity may be delayed in comparison to T cells *in vivo*. Additionally, most of the T cells in the TME have an exhausted and dysfunctional phenotype, and only a fraction are reactivated by the ICI treatment (8). Due to the comparatively low immune cell diversity and lack of systemic factors in an immunotumoroid model versus the *in vivo* TME, reactivation of exhausted cytotoxic T cells and the manifestation of detectable cytotoxicity likely requires some time. We therefore designed our immunotumoroid model to survive extended periods by optimizing the volume and components of the culture medium to support long-term survival (see Methods). **Figure 1** depicts a bladder immunotumoroid at multiple time points from day 0 until day 35. The tumor cell suspension is seeded on GravityPlus hanging drop plates on day -4 and the immunotumoroids are dropped into ULA plates on day 0. Even after 5 weeks of incubation without changing the media, the immunotumoroids are healthy with no sign of necrosis and apoptosis compared to the initial time points. By exchanging culture media, the immunotumoroids survived for over 65 days. This long-time survival was exhibited by all 5 cases that successfully formed immunotumoroids in this study, supporting the model’s suitability for ICI response evaluation.

### The immunotumoroid formation

Among 13 collected surgically resected tumor samples, 6 of them had extensive necrosis that prevented the formation of the tumor spheroid (immunotumoroid) in the hanging drop system. Immune checkpoint inhibitors (ICIs) are the blocking antibodies and do not have any cytotoxic effect on tumor cells by themselves; by blocking checkpoint proteins from binding with their ligands ICIs prevent the “off” signal from being sent, allowing the tumor-specific T cells to kill the tumor cells (9). Therefore, the prerequisite of any model to evaluate ICI response is the inclusion of tumor-specific T cells known as tumor infiltrated lymphocytes (TILs) (10–12). However, in our hands, an excess of TILs can prevent the formation of tumor spheroid. One of the collected tumor samples had ~ 40% TILs in the dissociated tumor cell suspension which was sufficient to prevent the formation of immunotumoroid.

The IHC staining of another tumor sample that successfully made tumor spheroid revealed less than 5% TILs in the primary tumor sample which made it ineligible as an immunotumoroid and was excluded from the study.

### The immunotumoroid model retains characteristics of the primary tumor

**Figure 2:**
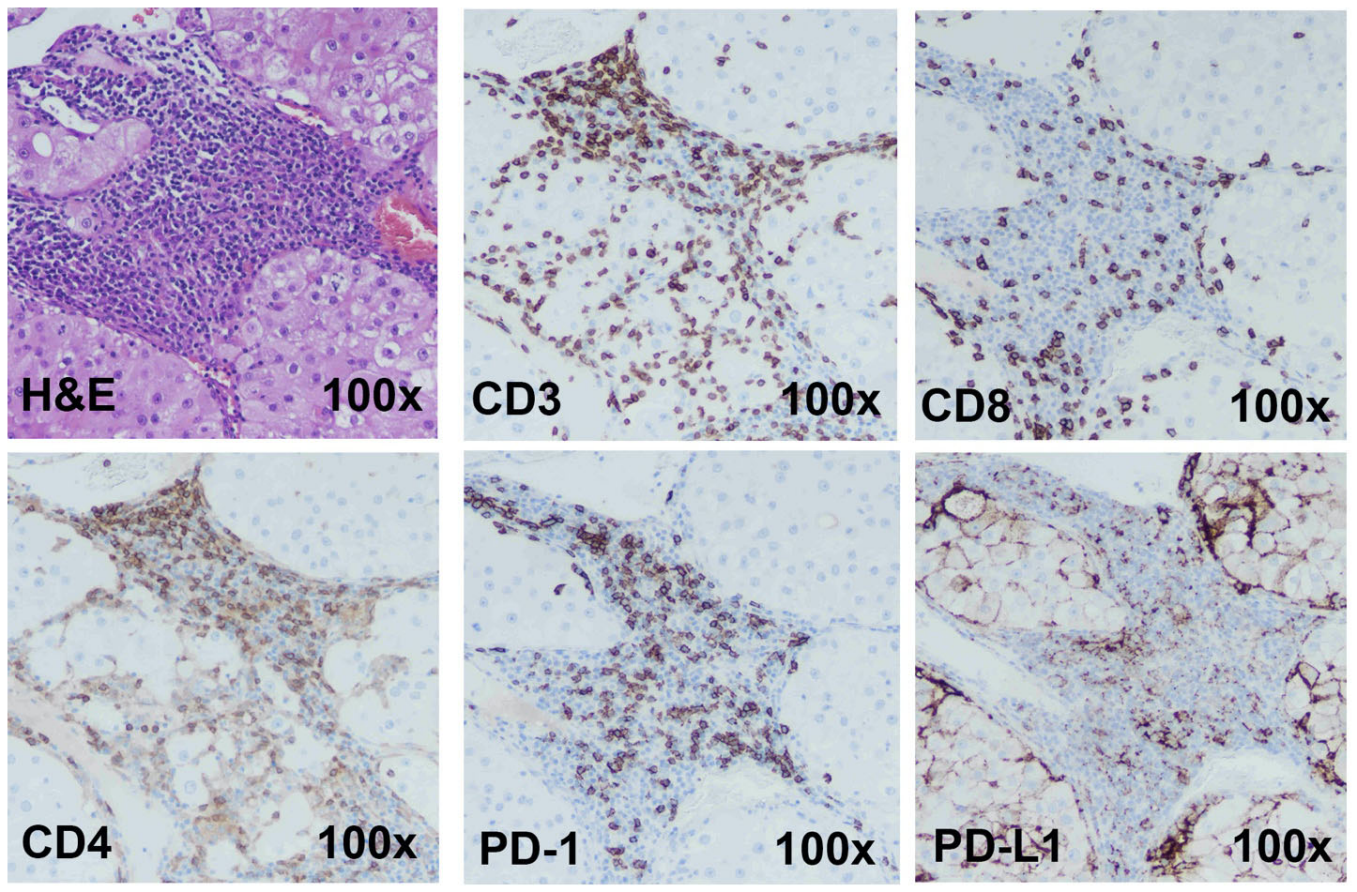
Immunohistochemistry staining shows Case 1 to be a candidate for ICI therapy The H&E, CD3 (total T cells), CD8 (cytotoxic T cells), CD4 (Helper or regulatory T cells) staining was done on a primary renal tumor (Case 1). The tumor has high T-cell infiltration. This tumor demonstrated high PD-1 and PD-L1 expression. Images are under 100x magnification.

**Figure 3:**
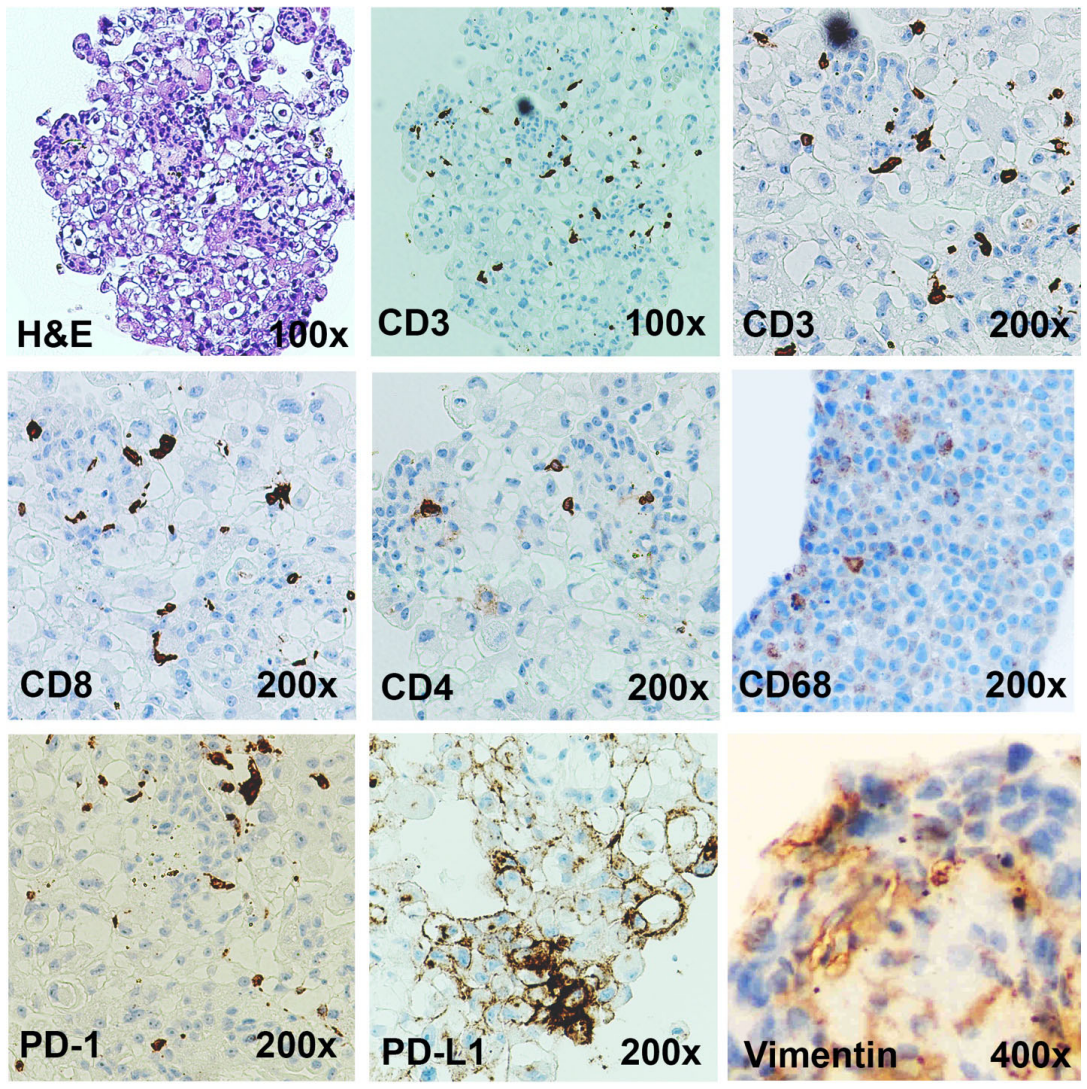
Immunotumoroids contain the immune cells and the stromal cells. The H&E, CD3 (total T cells), CD8 (cytotoxic T cells), CD4 (Helper or regulatory T cells), CD68 (TAMs), vimentin (CAFs), PD-1, and PD-L1 IHC staining was performed on the immunotumoroid prepared from renal tumor tissue (Case 1). The immunotumoroid successfully retained the cellularity and the expression profile (PD-1, PD-L1) of the original tumor tissue. Images are under 100x, 200x, and 400x magnification as labeled.

A model for ICI response evaluation must include immune and non-immune cells of TME that play crucial roles in the response to immunotherapy. Case 1 is a renal tumor with a high T cell (CD3^+^) infiltration. Cytotoxic CD8^+^ and helper/regulatory CD4^+^ T cells were detected by IHC, and the tumor shows high PD-1 and PD-L1 expression (**Figure 2**). A portion of the tumor was processed and cultured as immunotumoroids before IHC staining which shows that the resulting immunotumoroids retained the different subsets of T cells required for anti-tumor and ICI response evaluation, as well as PD-1 and PD-L1 expression (**Figure 3**).

The complex interactions between cellular and chemical components of the TME support tumor growth, development, and metastasis; fibroblasts (vimentin) and macrophages (CD68) are essential contributors to tumor development and also contribute to immunotherapy resistance (13–28). Tumor-associated macrophages (TAMs) are the most heterogenous immune cells in the TME, are critical for tumor growth (18, 19), and play a major role in ICI resistance (25–28). Secretion of epidermal growth factor (EGF), platelet-derived growth factor (PDGF), IL-6, IL-8, and IL-10 and activation of the Wnt/β-catenin signaling pathway by TAMs can directly induce tumor cell proliferation (29, 30), while secretion of metalloproteinases (MMPs), growth factors, and angiopoietin-1 promotes angiogenesis to provide nutrient and oxygen support (31, 32). Additionally, TAMs can decrease ICI efficacy by 1) directly blocking CD8^+^ T cells’ anti-tumor functions and tumor infiltration ability (25, 27, 33), 2) expressing alternate immune checkpoints (i.e., VISTA) or 3) sequestering ICI antibodies (25, 27, 34–36). The IHC staining confirms that the immunotumoroid model retained the TAMs from the primary tumor as presented in **Figure 3** by CD68 staining.

Cancer-associated fibroblasts (CAFs) are an essential component of the tumor stroma, with a major role in the physical support of tumor cells and enhancement of tumorigenesis (13–17). CAFs secrete various matrix proteins that play an essential role in the remodeling of tumor ECM (37, 38). Transforming growth factor-β (TGF-β) secreted by CAFs limits T cell infiltration into the tumor (39) while also downregulating the expression of MHC-II, CD80, and CD40 on the surface of dendritic cells in the TME, suppressing anti-tumor immunity (40). Additionally, CAFs promote differentiation and recruitment of Tregs, which (41) inhibit CD8^+^ T cells function and promote T cell exhaustion (42, 43). The Case 1 immunotumoroid model shows the presence of CAFs and TAMs as detected by IHC. Taken together, this data shows that the immunotumoroid model retains the cellularity and the immune checkpoint expression profile of the original tumor (**Figures 2**, **3**).

### The immunotumoroid model accurately predicts the clinical response to chemotherapy

To test the model’s functionality and sensitivity to therapeutic agents, the immunotumoroids were treated with chemotherapy drugs cisplatin, gemcitabine, and paclitaxel. Cell killing was quantified by Cytotox Red staining; preexisting dead cells are normalized to untreated controls, and autofluorescence is normalized through untreated-unstained controls.

**Figure 4:**
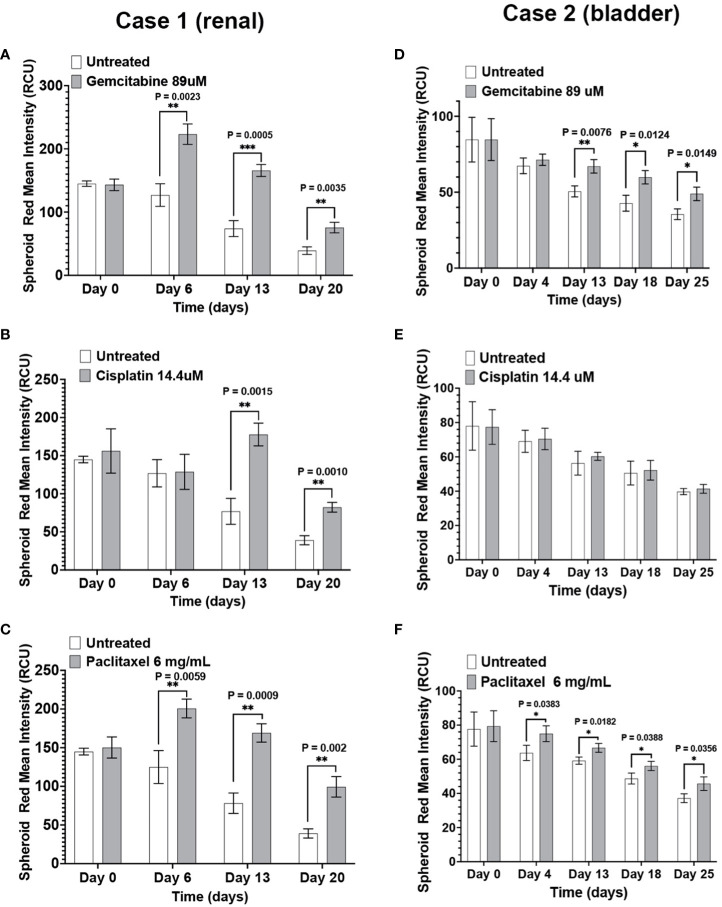
The immunotumoroid model responds to chemotherapy drugs: Renal (Case 1) and bladder (Case 2) immunotumoroids were treated with chemotherapy agents and their response was recorded at different time points. An increase in red fluorescence (Cytotox red staining) compared to the untreated control indicates cell killing. Case 1 was sensitive to gemcitabine **(A)**, cisplatin **(B)**, and paclitaxel **(C)**. Case 2 showed sensitivity to gemcitabine **(D)** and paclitaxel **(E)**, but resistance to cisplatin **(F)**. The results of other concentrations of drug treatment are shown in **Supplementary Figures 1A–F**. Statistical significance was determined by unpaired t-test; Error bars represent the mean ±SD of three independent experiments. *, P ≤ 0.05; **, P ≤ 0.01; ***, P ≤ 0.001. The intensity of red fluorescence decreases in later time points due to the degradation of dead cells and also loss of fluorescence intensity due to exposure to laser after scanning at each time point. Therefore, the fluorescent intensity of the treated immunotumoroids should be compared to the untreated controls at that time point but not the other time points.

The response of renal (Case 1) and bladder (Case 2) immunotumoroids to chemotherapy agents at different time points are shown in **Figure 4**. Case 1 shows sensitivity to gemcitabine, cisplatin, and paclitaxel (**Figures 4A–C**) compared to the non-treated controls; interestingly, the sensitivity to cisplatin becomes apparent at a later time point and was not detectable on day 6. These experiments were performed on 5 log concentrations and only the C_max_ concentrations are presented here (see **Supplementary Figures** for additional drug concentrations). Case 2 was also sensitive to gemcitabine and paclitaxel, but resistant to cisplatin (**Figures 4D–F**).

**Figure 5:**
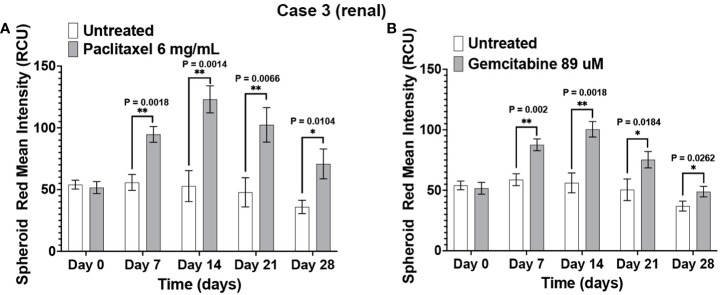
The immunotumoroid model reflects the patient’s clinical response to chemotherapy drugs. Case 3 immunotumoroids show sensitivity to paclitaxel **(A)** and, gemcitabine **(B)**. **Supplementary Figures 2A, B** show the response to other drug concentrations. Statistical significance was determined by unpaired t-test; Error bars represent the mean ±SD of three independent experiments. *, P ≤ 0.05; **, P ≤ 0.01.

The immunotumoroid drug screen results were compared to the patient clinical response to the drug when available. The patient in Case 3 responded to paclitaxel and later responded to gemcitabine. The drug screen results for Case 3 align with this clinical response; **Figure 5** shows that the immunotumoroid model responded well to both paclitaxel and gemcitabine beginning at day 7. Therefore, the immunotumoroid response to chemotherapeutic agents reflects the patient’s clinical response to these drugs. The patients of Cases 1, 4, and 5 did not receive any chemotherapy drug in their course of treatment. The patient from Case 2 had 1 cycle of gemcitabine and cisplatin but was not able to tolerate this due to renal impairment.

### Immunotumoroid response to ICIs

After confirming the immunotumoroid model’s suitability for chemotherapeutic drug screening, we evaluated the model’s response to ICI therapy using PD-1 and PD-L1 inhibitors pembrolizumab, nivolumab, and durvalumab. Pembrolizumab and nivolumab both target PD-1, but we tested both drugs to account for the possibility of different outcomes due to structural differences between the antibodies (44). Nivolumab and durvalumab were used in combination to evaluate possible synergistic effects observed in some cases from targeting both PD-1 and PD-L1 (45).

**Figure 6:**
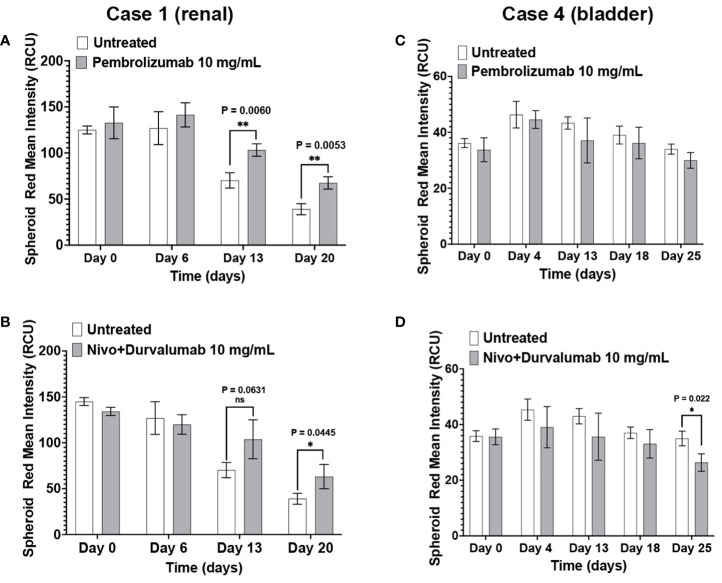
The immunotumoroid model responds to ICI treatment. Case 1 (renal cancer) and case 2 (bladder cancer) immunotumoroids were treated with pembrolizumab and a combination of nivolumab and durvalumab. While treatment with both pembrolizumab and nivolumab/durvalumab combination induced cytotoxicity in Case 1 at later time points **(A, B)**, neither of the ICI treatments induced cytotoxicity in Case 4 compared to untreated controls **(C, D)**. **Supplementary Figures 3A–D** show the response to other drug concentrations. Statistical significance was determined by unpaired t-test; Error bars represent the mean ±SD of three independent experiments. *, P ≤ 0.05; **, P ≤ 0.01; ns, not statistically significant.

Pembrolizumab and nivolumab/durvalumab combination therapy induced cytotoxicity in Case 1 (renal) immunotumoroids (**Figures 6A, B**). The response was apparent only at later time points (day 13 and day 20), which validates the need for long-term incubation of immunotumoroids in the context of ICI screening. Case 4 (bladder) immunotumoroids were resistant to both treatments compared to the untreated control (**Figures 6C, D**) Interestingly, Case 4 immunotumoroid viability increased after ICI treatment, which could be due to the hyperprogression phenomenon (46–51).

### Immunotumoroid response to Immune Checkpoint Inhibitors aligns with clinical data

**Figure 7:**
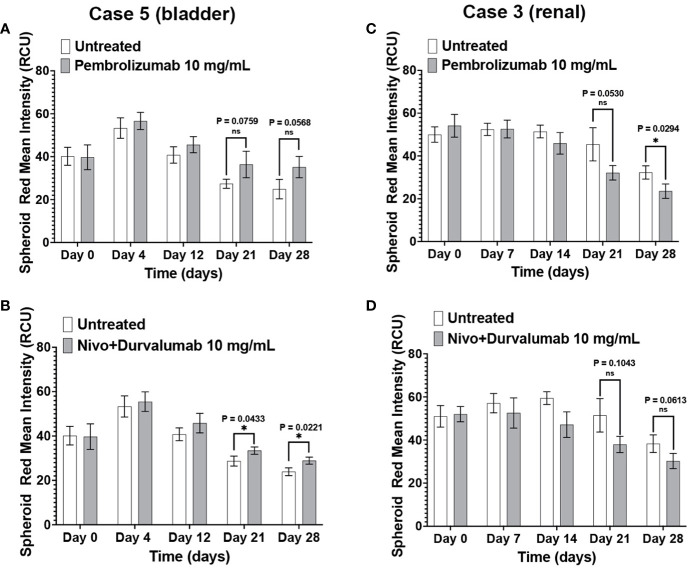
Clinical response validates the immunotumoroid response to ICIs. Pembrolizumab and a combination of nivolumab and durvalumab induce cell killing in Case 5 immunotumoroids at three-time points (52) compared to the untreated control **(A, B)**. The Case 5 patient responded clinically to treatment with pembrolizumab. Treatment of case 3 with ICIs improved the viability of the immunotumoroids instead of induction of cell death compared to the untreated control **(C, D)**. Clinically, the Case 3 patient progressed after 5 doses of nivolumab. **Supplementary Figures 4A–D** show the response to other drug concentrations. Statistical significance was determined by unpaired t-test; Error bars represent the mean ±SD of three independent experiments. *, P ≤ 0.05; ns, not statistically significant.

The patients in Case 3 and Case 5 have been treated with ICIs with different outcomes. Compared to the negative control, Case 5 immunotumoroids show sensitivity to both pembrolizumab and the combination of nivolumab and durvalumab (**Figures 7A, B**). This patient was diagnosed with clear cell renal cell carcinoma and underwent a year of pembrolizumab treatment after radical nephrectomy, and currently shows no evidence of disease. This clinical data correlates with the results of Case 5 immunotumoroid treatment with ICI.

Neither Pembrolizumab nor nivolumab/durvalumab therapies induced cell killing in Case 3 immunotumoroids (**Figures 7C, D**). Interestingly, immunotumoroid viability improved after ICI treatment compared to the untreated controls. The Case 3 patient had been diagnosed with renal carcinoma and progressed after receiving 5 doses of nivolumab. Therefore, the clinical response of Case 3 correlates with the immunotumoroid response to ICIs.

Clinical data and treatment outcomes of the cases are presented as a table in the **Supplementary Data.**

## Discussion

**Figure 8:**
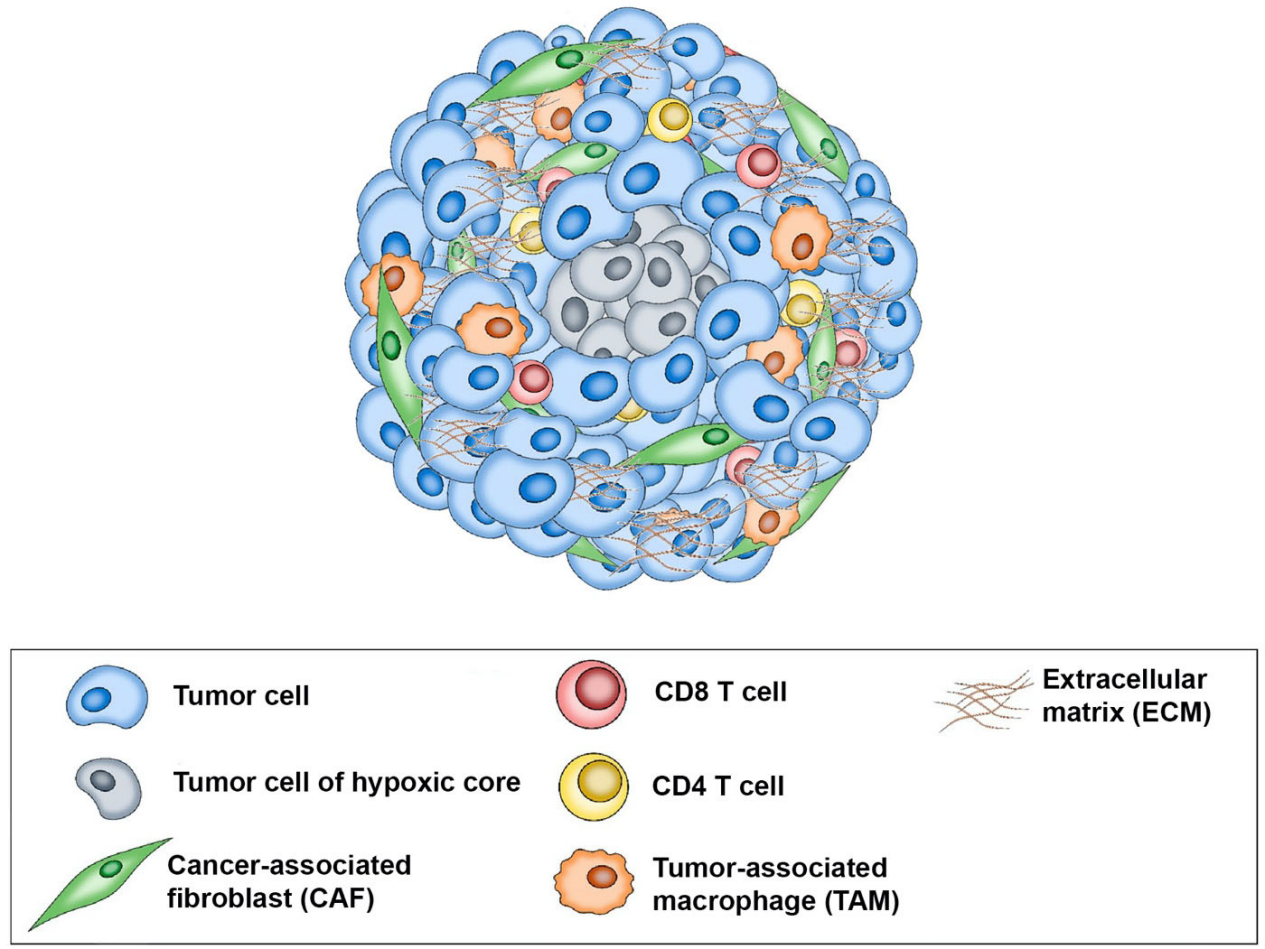
Schematic representation of the immunotumoroid model: After tissue dissociation, tumor cells, stromal cells, and tumor-infiltrating lymphocytes (TILs) reassemble into a spheroid microcancer (immunotumoroid) in a hanging drop culture. The hypoxic core of the immunotumoroid mimics the inner hypoxic region of a solid tumor, which may respond differently to drug treatment. Stromal cells including cancer-associated fibroblasts (CAFs) produce the extracellular matrix (ECM). Tumor-associated macrophages (TAMs) by secreting chemokine and cytokines support tumor cells and play a major role in ICI resistance. Immune checkpoint inhibitors (ICIs) can be added to the immunotumoroid culture, and their effects on immune cells (including CD8 and CD4 T cells) and their cytotoxicity toward tumor cells can be evaluated.

Predicting a patient’s response to treatment can help ensure that patients most likely to benefit from the drug will receive the drug while those unlikely to respond will not have delays in receiving effective treatment and can avoid the cost and potential side effects of ineffective therapy. Using an immunotumoroid model, the tumor’s response to immunotherapy drugs can be assessed in a controlled environment, providing valuable information about the potential effectiveness of a particular treatment for a specific patient. A course of immunotherapy drugs is time-consuming and expensive; immunotumoroid models offer an efficient and cost-effective way to evaluate the response to these treatments. The data obtained by testing different immunotherapies on the immunotumoroid model can aid the selection of the most promising options and more importantly, avoid the administration of drugs in patients that do not respond. **Figure 8** is a schematic representation of the immunotumoroid model.

While immunotumoroid models provide valuable insights into tumor biology and drug response, they cannot fully recapitulate the complexity and dynamic nature of the immune response in the human body. The immune system consists of a diverse population of immune cells with different functions and specificities; in a microcancer model, this diversity may be limited or absent. The absence of dendritic cells, natural killer cells, and/or myeloid cells, which interact with T cells and contribute to an effective immune response, can hinder the overall T cell reactivity observed *in vitro*. The body’s anti-cancer immune response is localized to the tumor site and is influenced by cytokines, chemokines, hormones, and regulatory feedback loops that can modulate immune cell activation, trafficking, and function. In a microcancer model, these systemic factors may be absent or not fully replicated, resulting in an incomplete representation of the complex immune regulation that occurs *in vivo*. Therefore, the efficiency of T cell reactions in microcancer models may be reduced compared to the *in vivo* setting. In consideration of these limitations when interpreting drug screen data and extrapolating findings to predict patient response, our immunotumoroid model was optimized for an extended time in culture.

There are inherent limitations, biases, and challenges in patient-derived microcancer models for cancer therapy, including size variability, lack of vascularization, tumor heterogeneity, biased selection of cells, biases in drug penetration, lack the complexity of the host immune responses, standardization of methods and protocols, and interpretation of results. 3D microcancers can vary significantly in size and morphology, which can affect their response to treatment and make it challenging to standardize experimental conditions. Microcancers lack a functional vascular system, which limits their ability to mimic the metabolic and drug diffusion properties of *in vivo* tumors. While microcancers can capture some aspects of tumor heterogeneity, they may not fully represent the genomic and phenotypic diversity found in primary tumors. The process of isolating and culturing cells to create microcancers can introduce biases, as only a subset of cells may survive and proliferate in the culture conditions. The way drugs penetrate and interact with the tumor can differ in microcancers compared to *in vivo* tumors. Microcancers may not fully capture the response of the immune system to immunotherapy, as they lack the complexity of *in vivo* immune responses and the potential effects of the host immune system on tumor growth. There is currently a lack of standardization in the methods and protocols used to generate and culture microcancers, which can lead to variability and inconsistent results. It can be challenging to interpret the results of organoid experiments, as the responses observed *in vitro* may not always translate to *in vivo* settings due to the limitations mentioned above.

Traditional chemotherapeutic agents are ideal for microcancer model performance evaluation. Chemotherapy remains the standard treatment approach for many cancers, and evaluating the efficacy of a microcancer model in predicting response to these drugs can be valuable. The diverse range of available chemotherapeutic agents represents different classes of drugs with different mechanisms of action, each validated in different cancer types; this diversity improves model performance assessment by enabling careful selection of drugs clinically approved for the model’s specific tumor type. In some cases, the availability of clinical data allows us to validate the predictions made by the microcancer model through correlation with clinical responses. Although our study is small, our data suggests that an immunotumoroid model has the potential to predict the patient’s response. The microcancer model must have T cells present. T cells play a central role in the anti-cancer immune response, as they are responsible for recognizing tumor antigens and eliminating tumor cells. ICI therapy targets T cells for reactivation, enhancing anti-tumor immune responses. Our immunotumoroid model incorporates T cells from the original tumor to increase the chance of including tumor-specific T cells, which are present in TILs at around 200 times the frequency of that found in PBMCs (10–12); this makes our model more physiologically relevant for evaluating the anti-tumor T cell response upon ICI treatment. The tumor-specific T cells in each immunotumoroid may or may not be responsive to the ICI treatment based on the immunosuppressive mechanisms they previously encountered in their particular TME.

It is worth noting that one should not expect the same level of cell killing from ICIs as from chemotherapeutic agents in an *ex vivo* microcancer model due to differences in the drugs’ mechanisms of action. ICIs enhance the activity of the immune system by blocking certain checkpoints that regulate immune responses against cancer cells; in contrast, chemotherapeutic agents directly kill both cancer cells and non-cancer cells. The cytotoxic effects of ICIs typically manifest over a longer time compared to chemotherapeutic agents. While chemotherapeutic agents act rapidly and with direct cytotoxicity on different cell populations regardless of individual characteristics, ICIs work by stimulating the immune system’s response against cancer cells; this involves the activation and proliferation of immune cells, which may extend the time required for full effect. Additionally, cells within a tumor exhibit significant heterogeneity that can result in a mixed ICI response timeline due to various factors, including the level of checkpoint expression in each cell population. Our data illustrates this difference in response time as Case 1 (**Figures 6A, B**) and Case 5 (**Figures 7A, B**) immunotumoroids become responsive to ICIs only after 13 and 21 days of treatment, while immunotumoroids responded to chemotherapy drugs within 4 -7 days of treatment (**Figures 4**, **5**). Interestingly, the cytotoxic effect of ICIs on Case 5 and Case 3 immunotumoroids aligned with each patient’s clinical response; this strongly supports the immunotumoroid model’s capability to predict patient response to ICI therapy.

In Case 3 (**Figures 7C, D**) and Case 4 (**Figure 6D**), the immunotumoroid viability increased after ICI treatment, possibly the result of hyperprogression. Hyperprogressive disease (HPD) refers to the accelerated tumor growth observed in some cancer patients after treatment with ICIs (46–51). The HPD incidence rate is highly variable, ranging from 4 to 29% among different cancer types, and it may occur through several mechanisms. ICI-driven activation and expansion of PD-1^+^ regulatory T cells could support tumor growth and cultivate an immunosuppressive TME (48, 49). HPD could also be caused by ICI-driven activation of PD-1^+^ M2-TAMs, the release of protumor cytokines by activated Th17, and/or subclonal populations of cancer cells carrying mutations that result in oncogenic pathway activation by PD-1/PD-L1 axis blockade (48, 53).

In conclusion, we present a microcancer model designed to predict an individual tumor’s responses to chemotherapy and ICI treatment. This study demonstrates feasibility on a small number of cases, and additional studies with large case numbers are required including correlation with clinical response. Our immunotumoroid model shows the potential for a useful tool for the preclinical evaluation of ICIs and patient response prediction and holds promise for the evaluation of novel single-agent and combination therapy candidates. The need for combination therapies to overcome monotherapy resistance often limits ICI utility for many tumor types. We are optimizing our model to become eligible for the evaluation of combinational therapy in cases that are resistant to monotherapy. Currently, our ongoing studies are recruiting larger populations for this study and evaluation of other ICIs (e.g., CTLA-4, LAG-3, TIM-3) and assessment of new therapeutic approaches using multiple ICIs in combination or with targeted therapies. To correlate immunotumoroid model predictions with clinical responses we plan to collaborate with oncologists, potentially in a clinical trial setting, to validate the predictive value of the immunotumoroid model for immunotherapeutic response. Correlating the response of the immunotumoroid to immunotherapy in the model with the patient’s response to the same drug will allow us to better understand the translatability of the immunotumoroid model.

## Data availability statement

The original contributions presented in the study are included in the article/**Supplementary Material.** Further inquiries can be directed to the corresponding authors.

## Ethics statement

The studies involving humans were approved by The Ethics Committee of Mayo Clinic. The studies were conducted in accordance with the local legislation and institutional requirements. The participants provided their written informed consent to participate in this study.

## Author contributions

AP: Conceptualization, Data curation, Formal analysis, Investigation, Methodology, Validation, Visualization, Writing – original draft, Writing – review & editing. JC: Funding acquisition, Methodology, Supervision, Writing – review & editing. AF: Writing – review & editing. BL: Writing – review & editing. GV: Conceptualization, Funding acquisition, Supervision, Writing – review & editing.
