# Supplementary material for: Evaluation of tumor response to immune checkpoint inhibitors by a 3D immunotumoroid model
Source: Front Immunol. 2024 Mar 28;15:1356144. doi: 10.3389/fimmu.2024.1356144 (PMC11007648; doi:10.3389/fimmu.2024.1356144)
Supplement: Supplementary file 1 [file Table_1.docx]

**Supplemental Table 1. Clinical data and treatment outcomes of the cases:**

| **Case** | **Patient Characteristics** | **Treatment** | **Adjuvant Therapy/Clinical Response** | **Immunotumoroid Response** |
| --- | --- | --- | --- | --- |
| 1 | 47y, Male  Papillary renal cell carcinoma | Radical nephrectomy | None Given | **Responsive:** cisplatin, gemcitabine, paclitaxel, pembrolizumab, nivolumab+durvalumab  **Resistant:** none |
| 2 | 66y, Male  Bladder cancer | Radical cystectomy | 1 cycle gemcitabine + cisplatin; not tolerated | **Responsive:** gemcitabine, paclitaxel  **Resistant:** cisplatin |
| 3 | 59y, Female  Clear cell renal carcinoma | Radical nephrectomy | Paclitaxel, followed by gemcitabine. Partial response then progression. Nivolumab, progression after 5 cycles | **Responsive:** none  **Resistant:** pembrolizumab, nivolumab+durvalumab |
| 4 | 82y, Male  Bladder cancer | cystectomy | None | **Responsive:** cisplatin, gemcitabine, paclitaxel  **Resistant:** pembrolizumab, nivolumab+durvalumab |
| 5 | 71y, Male  Clear cell renal carcinoma | Radical nephrectomy | Responded to pembrolizumab | **Responsive:** pembrolizumab, nivolumab+durvalumab  **Resistant:** none |
