# Supplementary material for: Evaluation of tumor response to immune checkpoint inhibitors by a 3D immunotumoroid model
Source: Front Immunol. 2024 Mar 28;15:1356144. doi: 10.3389/fimmu.2024.1356144 (PMC11007648; doi:10.3389/fimmu.2024.1356144)
Supplement: Supplementary file 2 [file DataSheet_1.docx]

**SUPPLEMENTAL FIGURES**


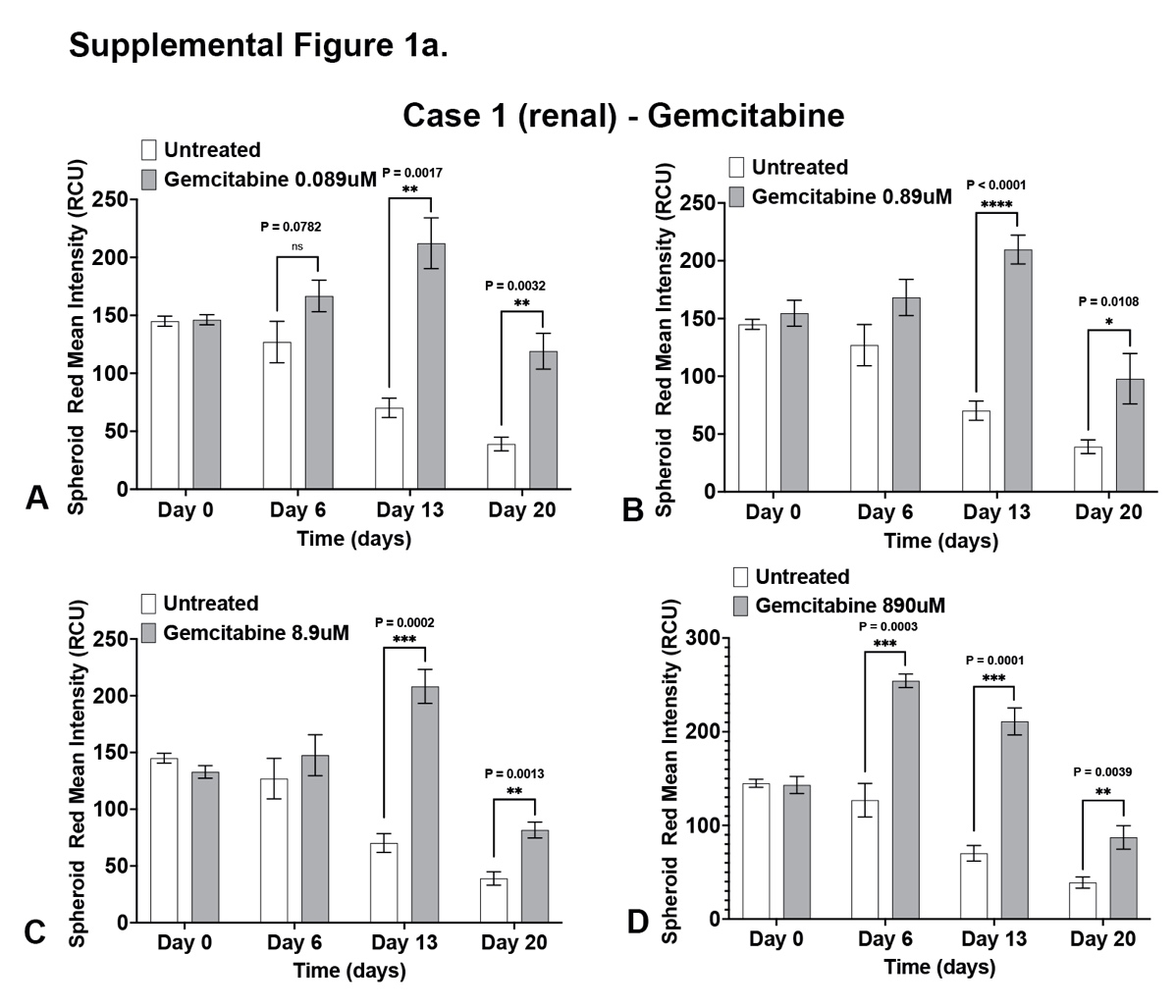


**Supplemental Figure 1a. The renal (Case 1) immunotumoroid responds to gemcitabine**: An increase in red fluorescence (Cytotox red staining) compared to the untreated control indicates cell killing. Case 1 was sensitive to gemcitabine. Statistical significance was determined by unpaired t-test; Error bars represent the mean ±SD of three independent experiments. *, P ≤ 0.05; **, P ≤ 0.01; ***, P ≤ 0.001; ****, P ≤ 0.0001.


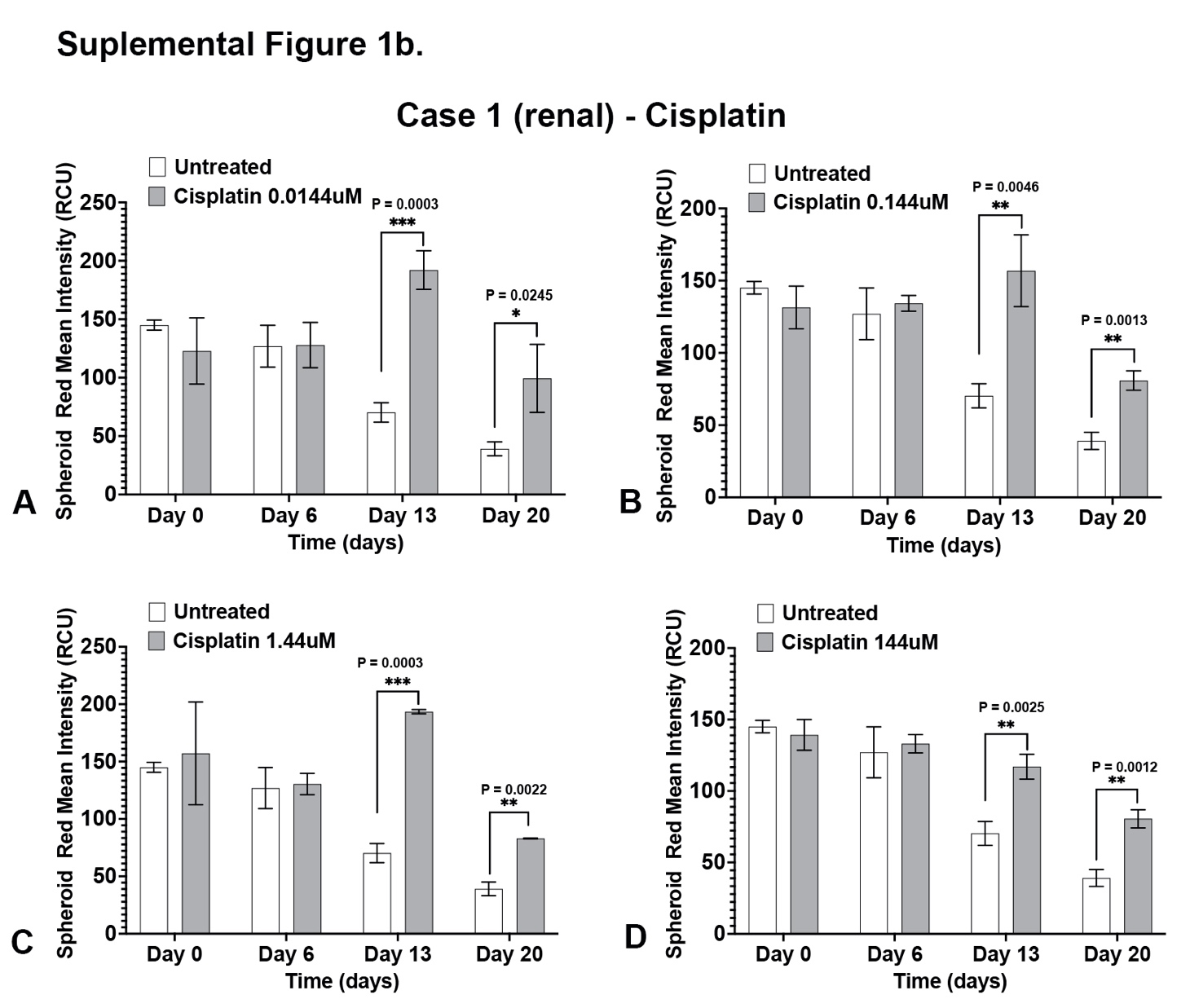


**Supplemental Figure 1b. The renal (Case 1) immunotumoroid responds to cisplatin**: An increase in red fluorescence (Cytotox red staining) compared to the untreated control indicates cell killing. Case 1 was sensitive to cisplatin. Statistical significance was determined by unpaired t-test; Error bars represent the mean ±SD of three independent experiments. *, P ≤ 0.05; **, P ≤ 0.01; ***, P ≤ 0.001.


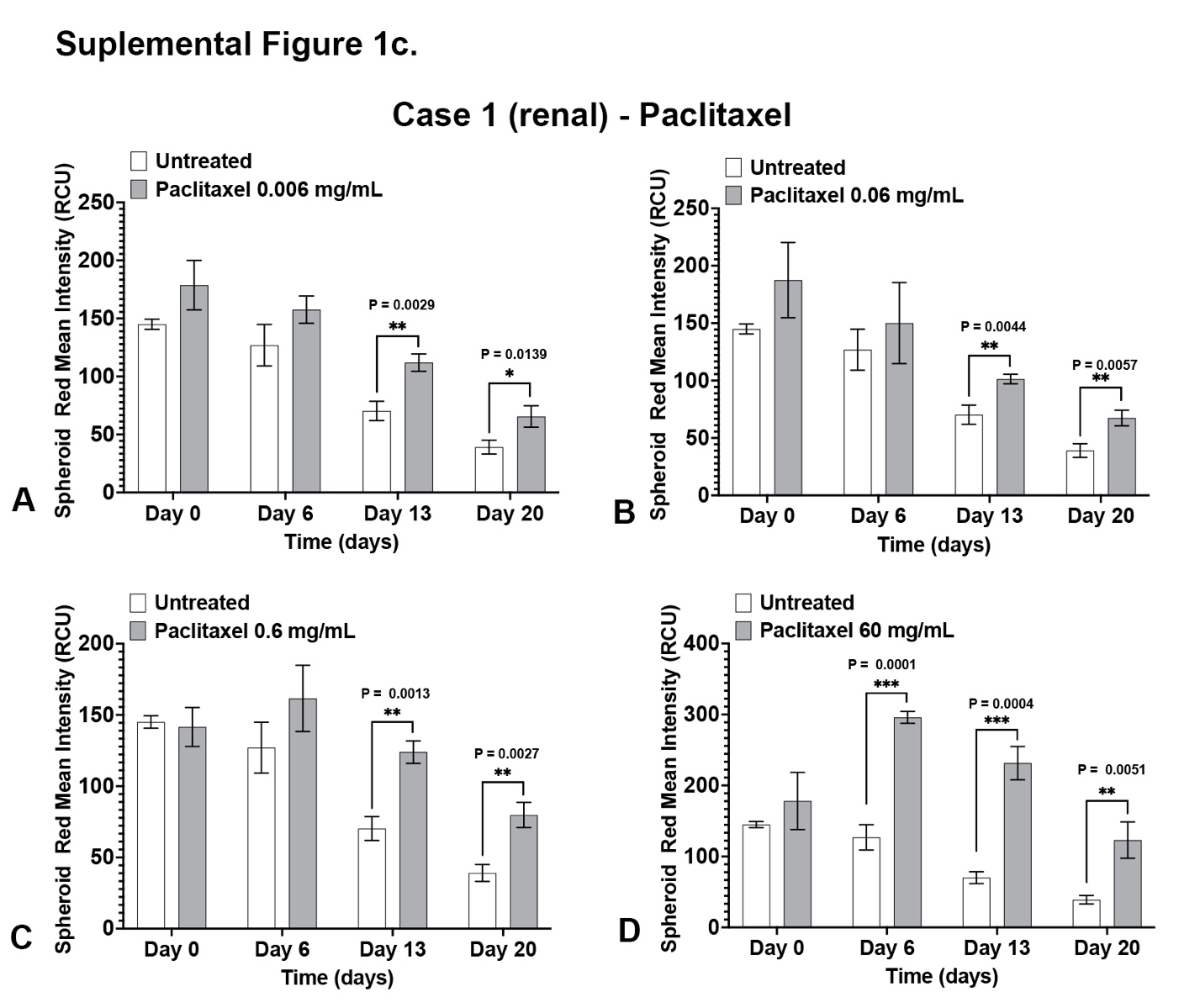


**Supplemental Figure 1c. The renal (Case 1) immunotumoroid responds to paclitaxel**: An increase in red fluorescence (Cytotox red staining) compared to the untreated control indicates cell killing. Case 1 was sensitive to paclitaxel. Statistical significance was determined by unpaired t-test; Error bars represent the mean ±SD of three independent experiments. *, P ≤ 0.05; **, P ≤ 0.01; ***, P ≤ 0.001.


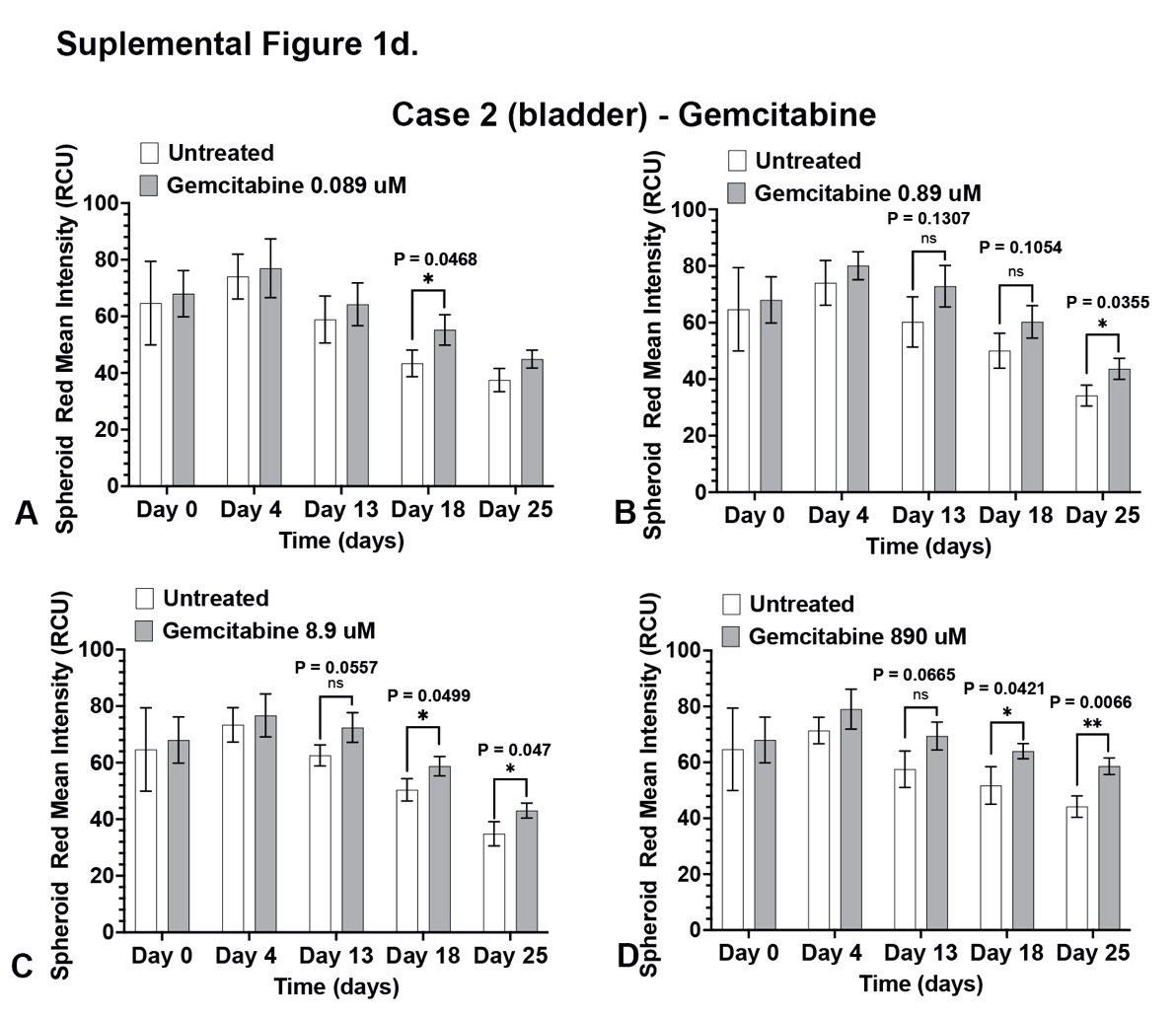


**Supplemental Figure 1d. The bladder (Case 2) immunotumoroid responds to gemcitabine**: An increase in red fluorescence (Cytotox red staining) compared to the untreated control indicates cell killing. Case 2 showed sensitivity to gemcitabine. Statistical significance was determined by unpaired t-test; Error bars represent the mean ±SD of three independent experiments. *, P ≤ 0.05; **, P ≤ 0.01, "ns" not statistically significant.


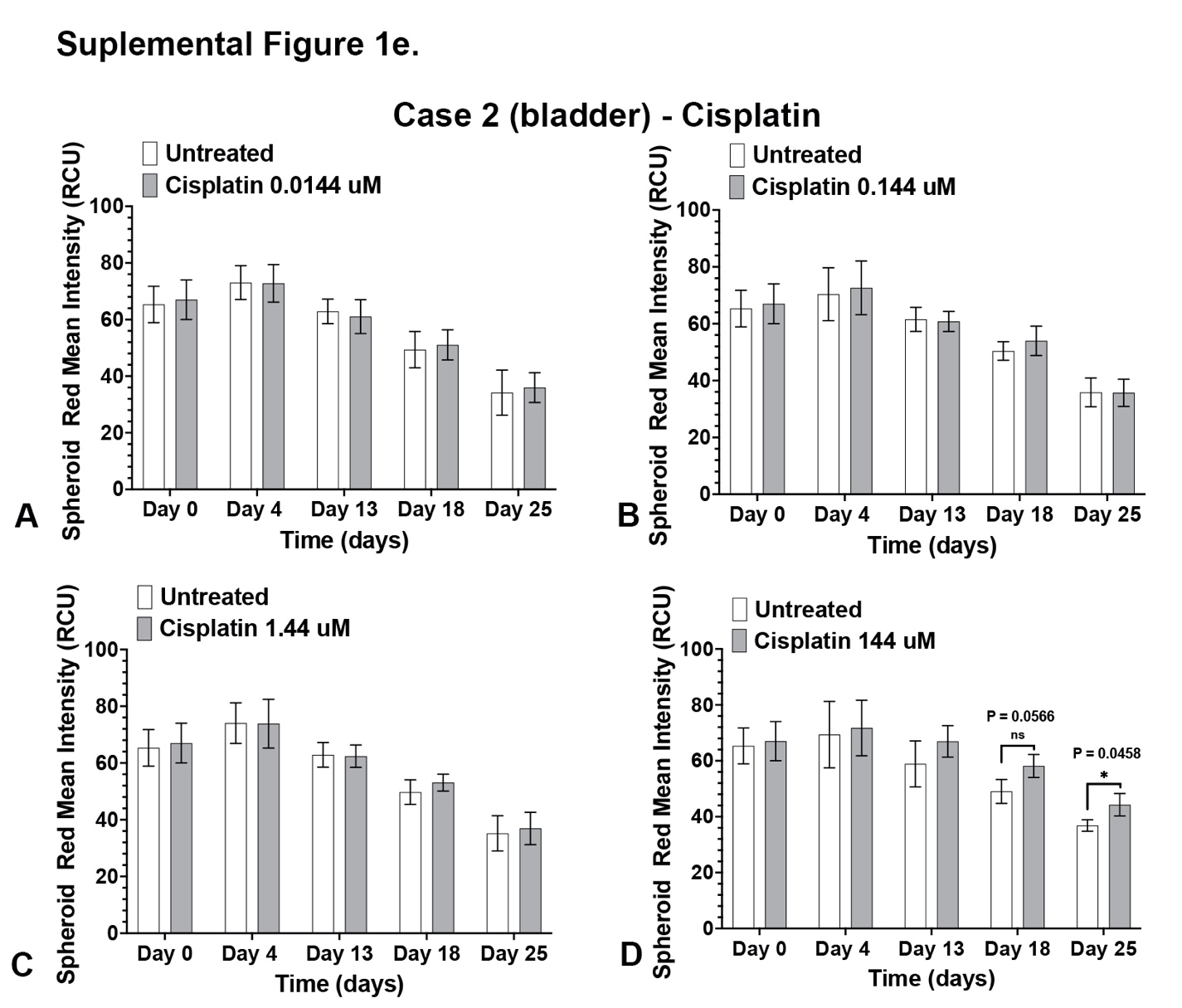


**Supplemental Figure 1e. The bladder (Case 2) immunotumoroid responds to cisplatin**: An increase in red fluorescence (Cytotox red staining) compared to the untreated control indicates cell killing. Case 2 showed resistance to cisplatin. Statistical significance was determined by unpaired t-test; Error bars represent the mean ±SD of three independent experiments. *, P ≤ 0.05, "ns" not statistically significant.


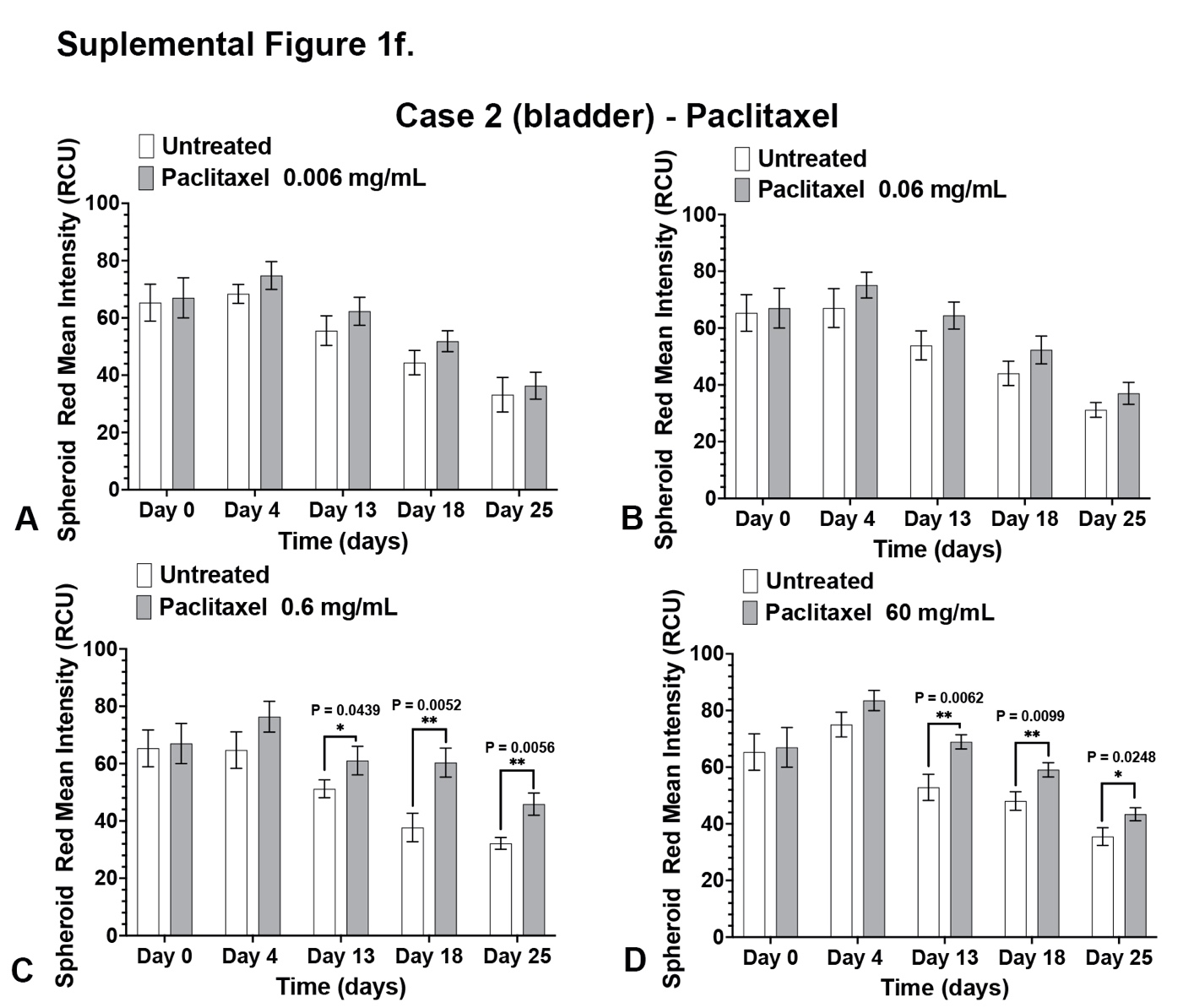


**Supplemental Figure 1f. The bladder (Case 2) immunotumoroid responds to paclitaxel**: An increase in red fluorescence (Cytotox red staining) compared to the untreated control indicates cell killing. Case 2 showed sensitivity to paclitaxel. Statistical significance was determined by unpaired t-test; Error bars represent the mean ±SD of three independent experiments. *, P ≤ 0.05; **, P ≤ 0.01.


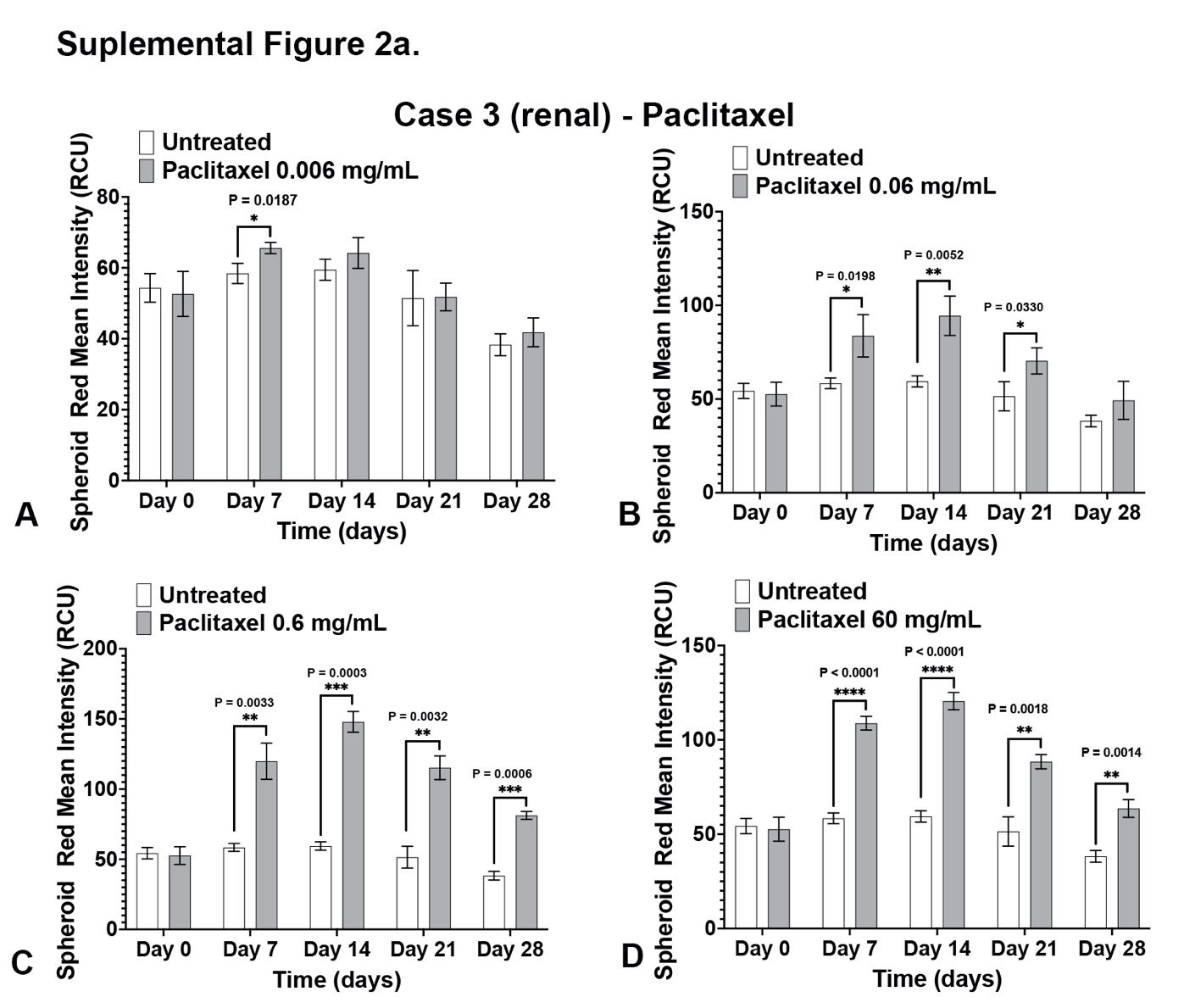


**Supplemental Figure 2a. The immunotumoroid model reflects the patient’s clinical response to chemotherapy drugs.** Case 3 immunotumoroids show sensitivity to paclitaxel. Statistical significance was determined by unpaired t-test; Error bars represent the mean ±SD of three independent experiments. *, P ≤ 0.05; **, P ≤ 0.01; ***, P ≤ 0.001; ****, P ≤ 0.0001.


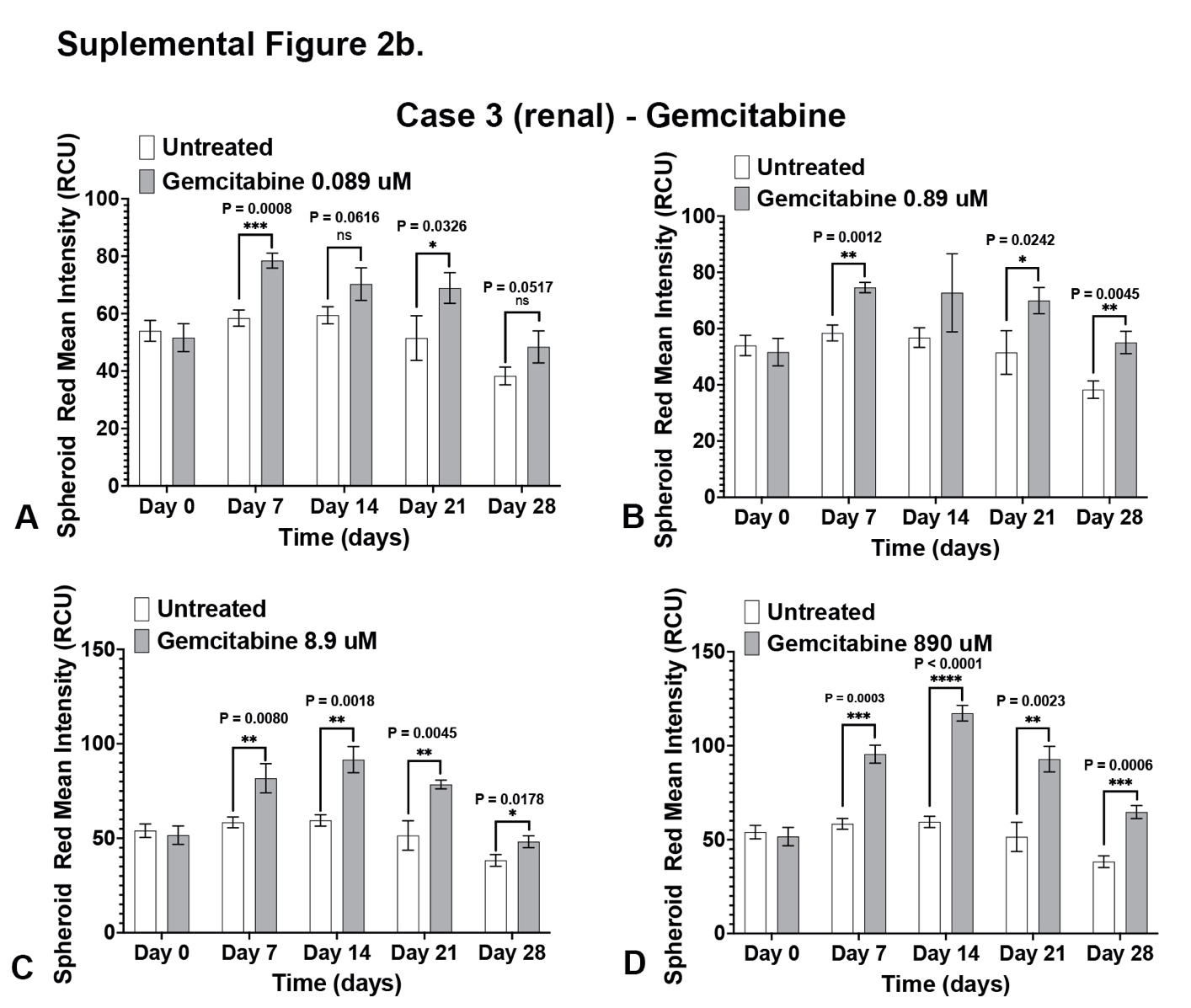


**Supplemental Figure 2b. The immunotumoroid model reflects the patient’s clinical response to chemotherapy drugs.** Case 3 immunotumoroids show sensitivity to gemcitabine. Statistical significance was determined by unpaired t-test; Error bars represent the mean ±SD of three independent experiments. *, P ≤ 0.05; **, P ≤ 0.01; ***, P ≤ 0.001; ****, P ≤ 0.0001, "ns" not statistically significant.


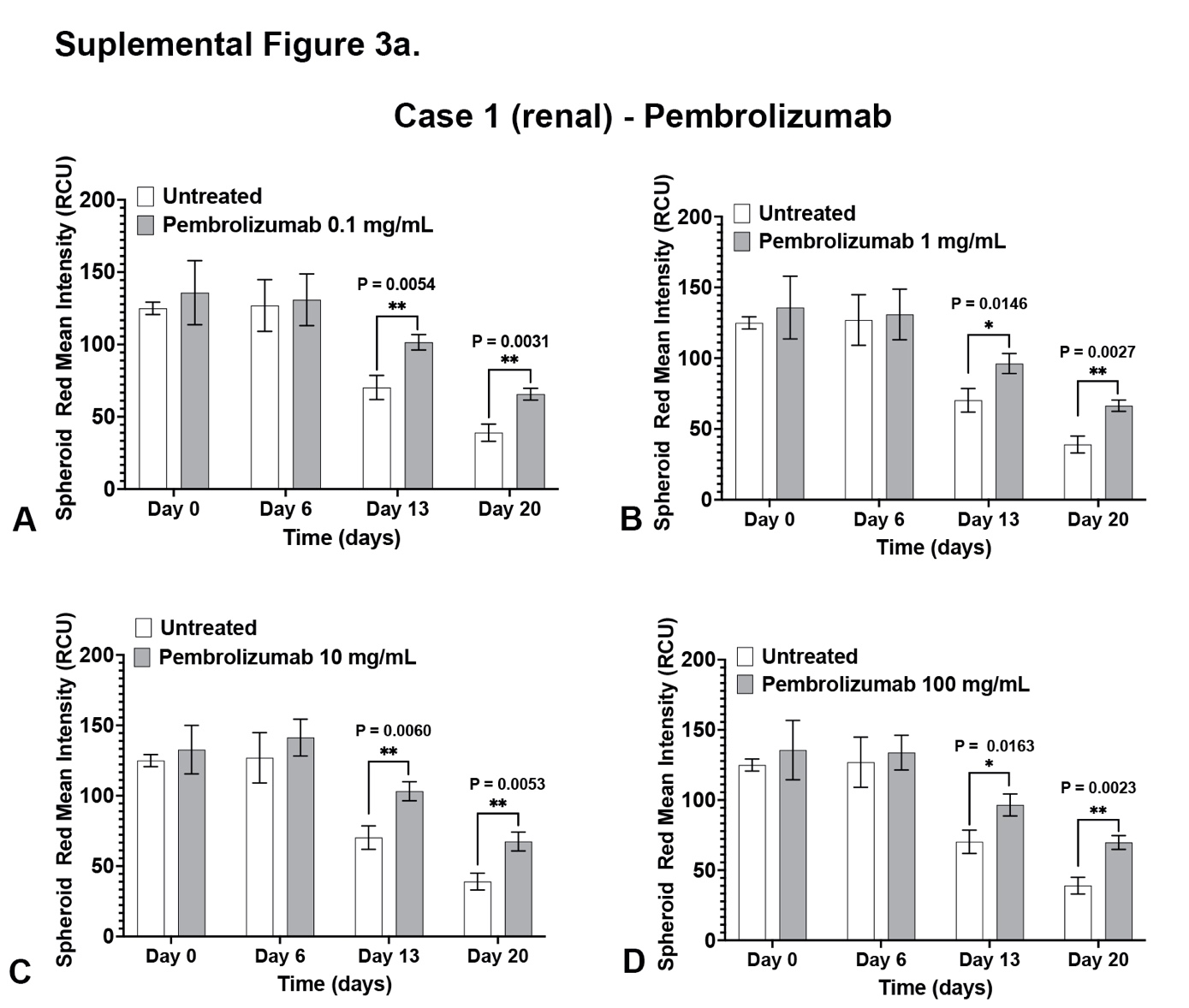


**Supplemental Figure 3a. The immunotumoroid model responds to ICI therapy.** Case 1 (renal cancer) immunotumoroids were treated with pembrolizumab. Treatment with pembrolizumab induced cytotoxicity in Case 1 at later time points compared to untreated controls. Statistical significance was determined by unpaired t-test; Error bars represent the mean ±SD of three independent experiments. *, P ≤ 0.05; **, P ≤ 0.01.


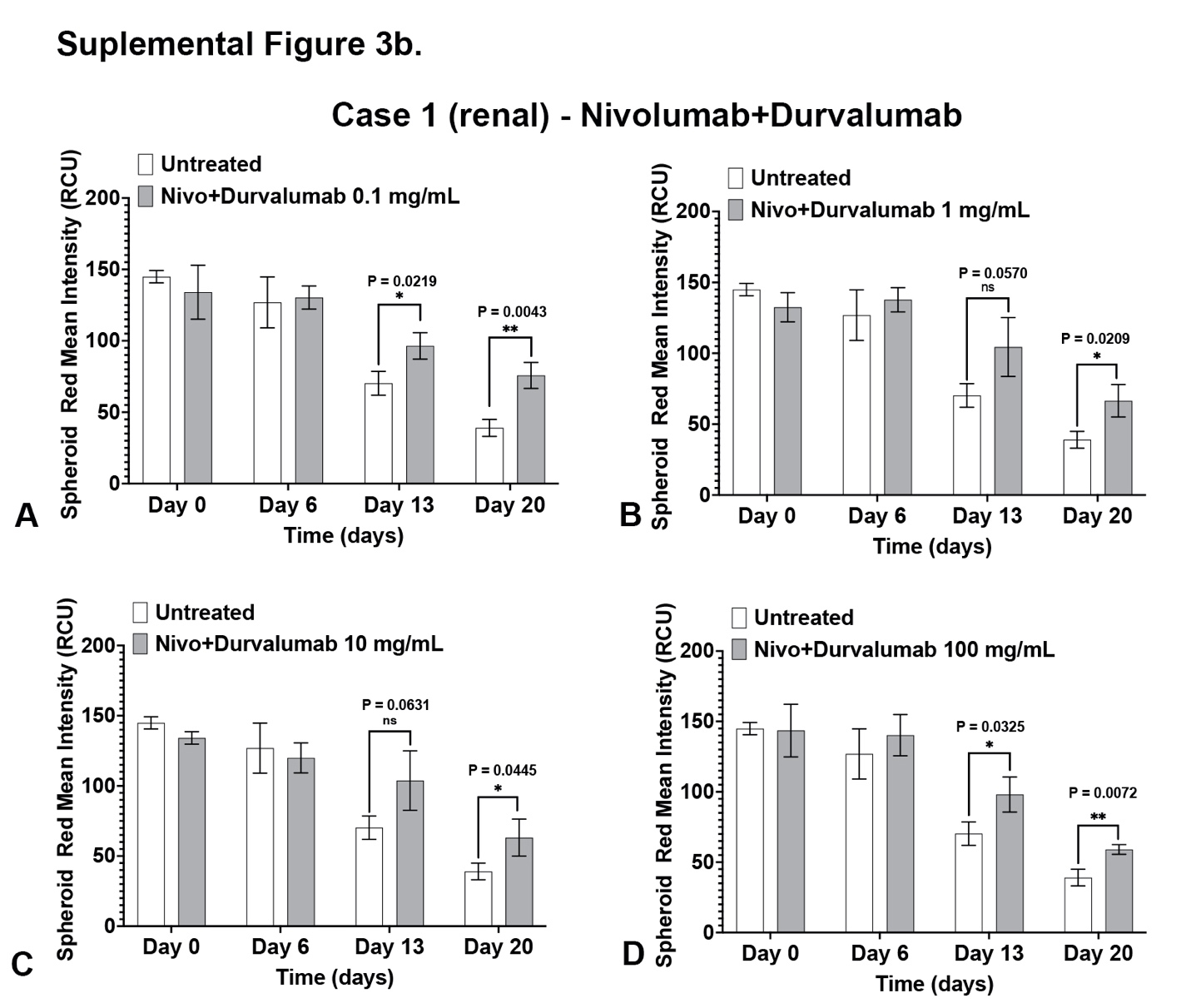


**Supplemental Figure 3b. The immunotumoroid model responds to ICI therapy.** Case 1 (renal cancer) immunotumoroids were treated with a combination of nivolumab and durvalumab. The combination of nivolumab and durvalumab induced cytotoxicity in Case 1 at later time points compared to untreated controls. Statistical significance was determined by unpaired t-test; Error bars represent the mean ±SD of three independent experiments. *, P ≤ 0.05; **, P ≤ 0.01, "ns" not statistically significant.


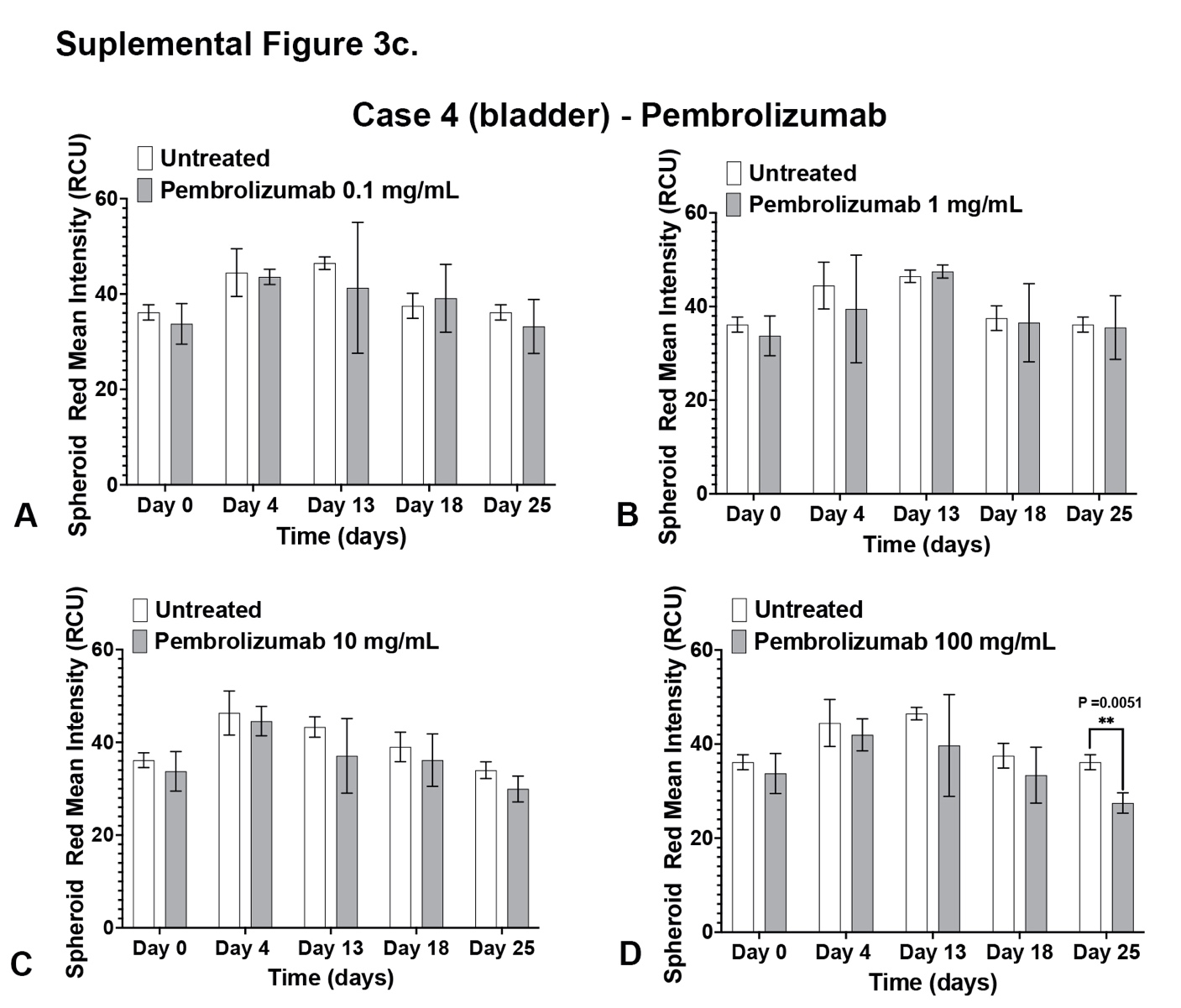


**Supplemental Figure 3c. The immunotumoroid model responds to ICI therapy.** Case 4 (bladder cancer) immunotumoroids were treated with pembrolizumab. Treatment with pembrolizumab did not induce cytotoxicity in Case 4. Statistical significance was determined by unpaired t-test; Error bars represent the mean ±SD of three independent experiments. **, P ≤ 0.01.


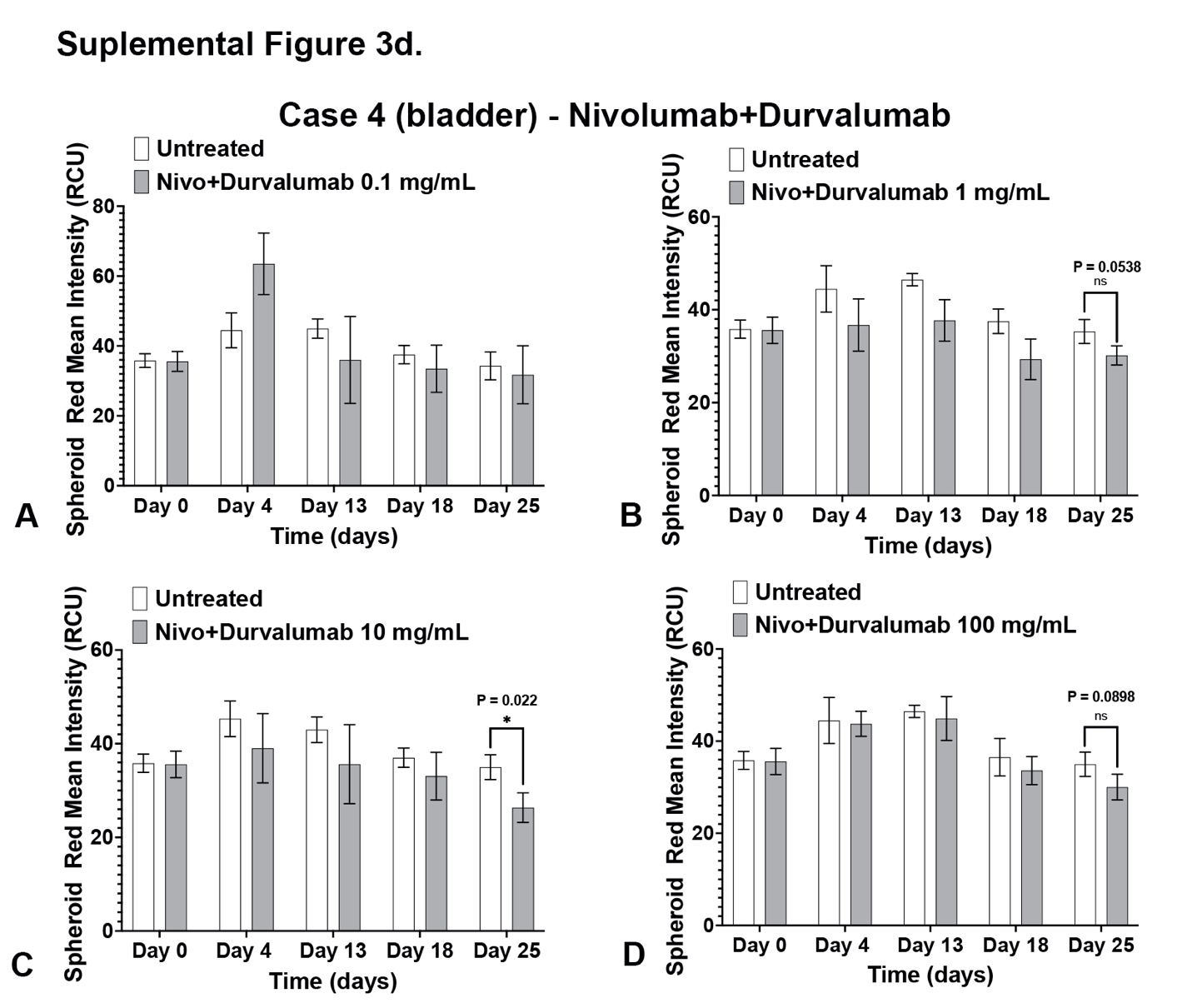


**Supplemental Figure 3d. The immunotumoroid model responds to ICI therapy.** Case 4 (bladder cancer) immunotumoroids were treated with a combination of nivolumab and durvalumab. Treatment with the combination of nivolumab and durvalumab did not induce cytotoxicity in Case 4. Statistical significance was determined by unpaired t-test; Error bars represent the mean ±SD of three independent experiments. *, P ≤ 0.05, "ns" not statistically significant.


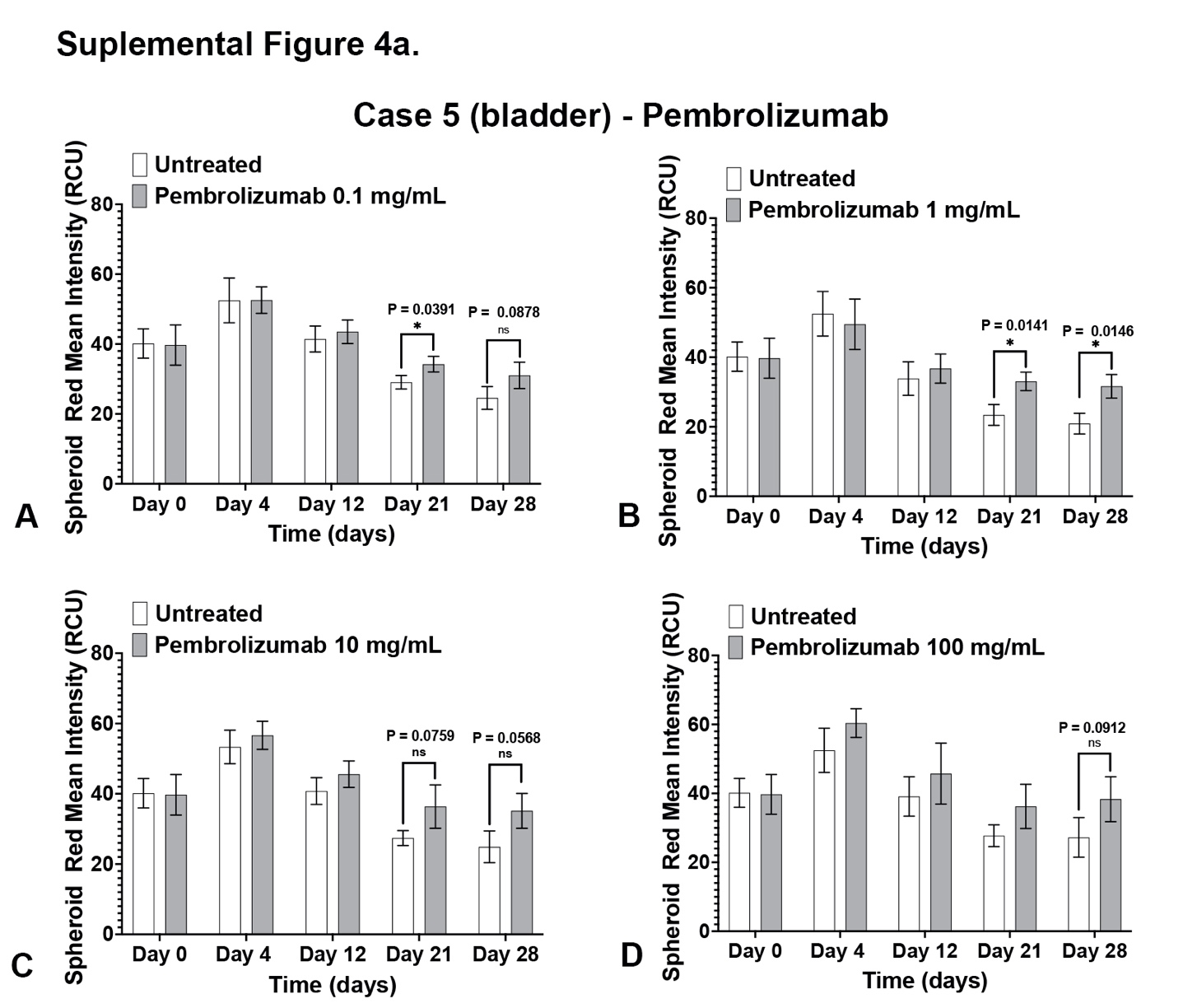


**Supplemental Figure 4a. Clinical response validates the immunotumoroid response to ICIs (Case 5).** Pembrolizumab induced cell killing in Case 5 immunotumoroids at three time points (day 12, day 21, and day 28) compared to the untreated control. The Case 5 patient responded clinically to treatment with pembrolizumab. Statistical significance was determined by unpaired t-test; Error bars represent the mean ±SD of three independent experiments. *, P ≤ 0.05, "ns" not statistically significant.


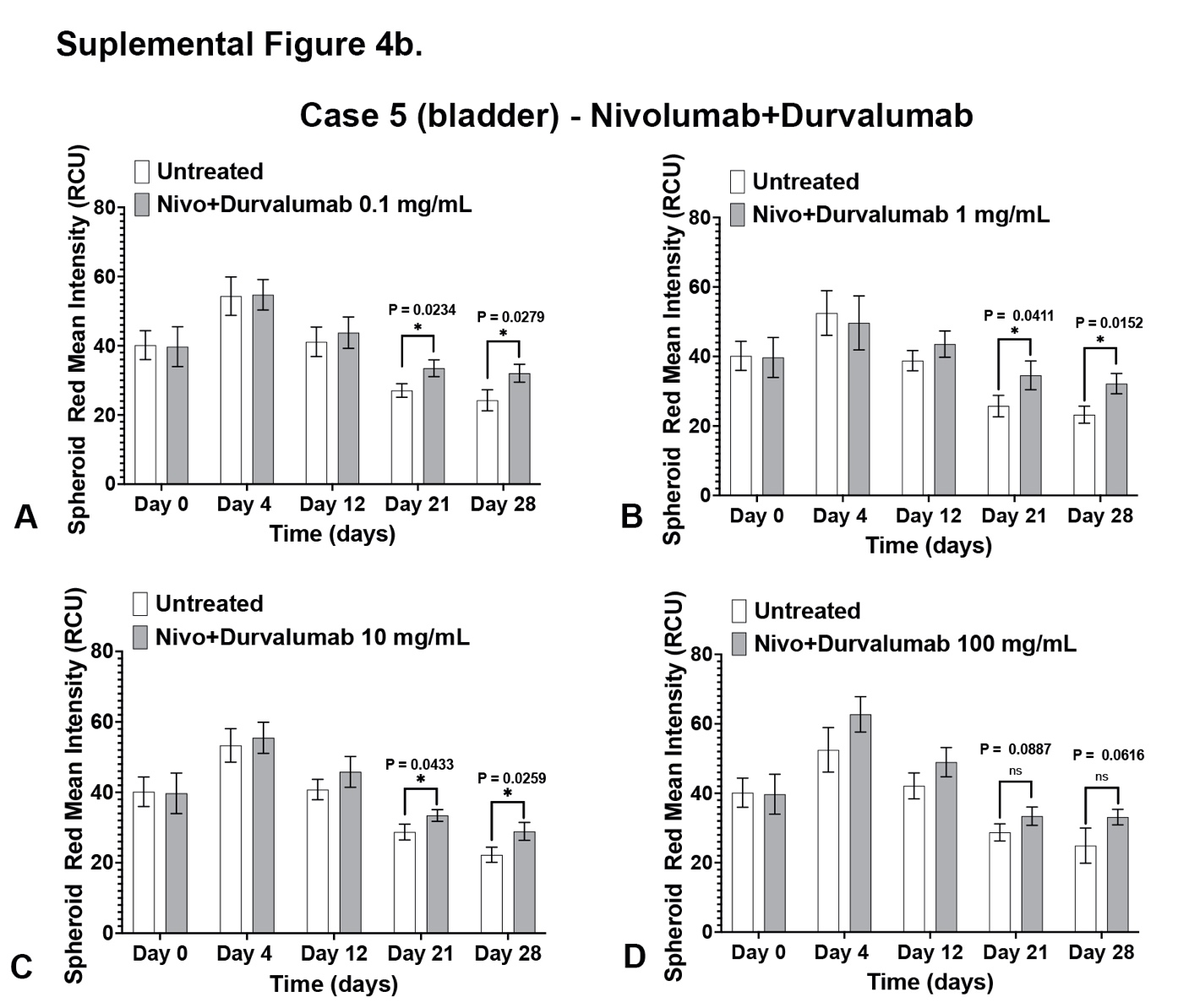


**Supplemental Figure 4b. Clinical response validates the immunotumoroid response to ICIs (Case 5).** A combination of nivolumab and durvalumab induced cell killing in Case 5 immunotumoroids at three-time points (day 12, day 21, and day 28) compared to the untreated control. The Case 5 patient responded clinically to treatment with pembrolizumab. Statistical significance was determined by unpaired t-test; Error bars represent the mean ±SD of three independent experiments. *, P ≤ 0.05, "ns" not statistically significant.


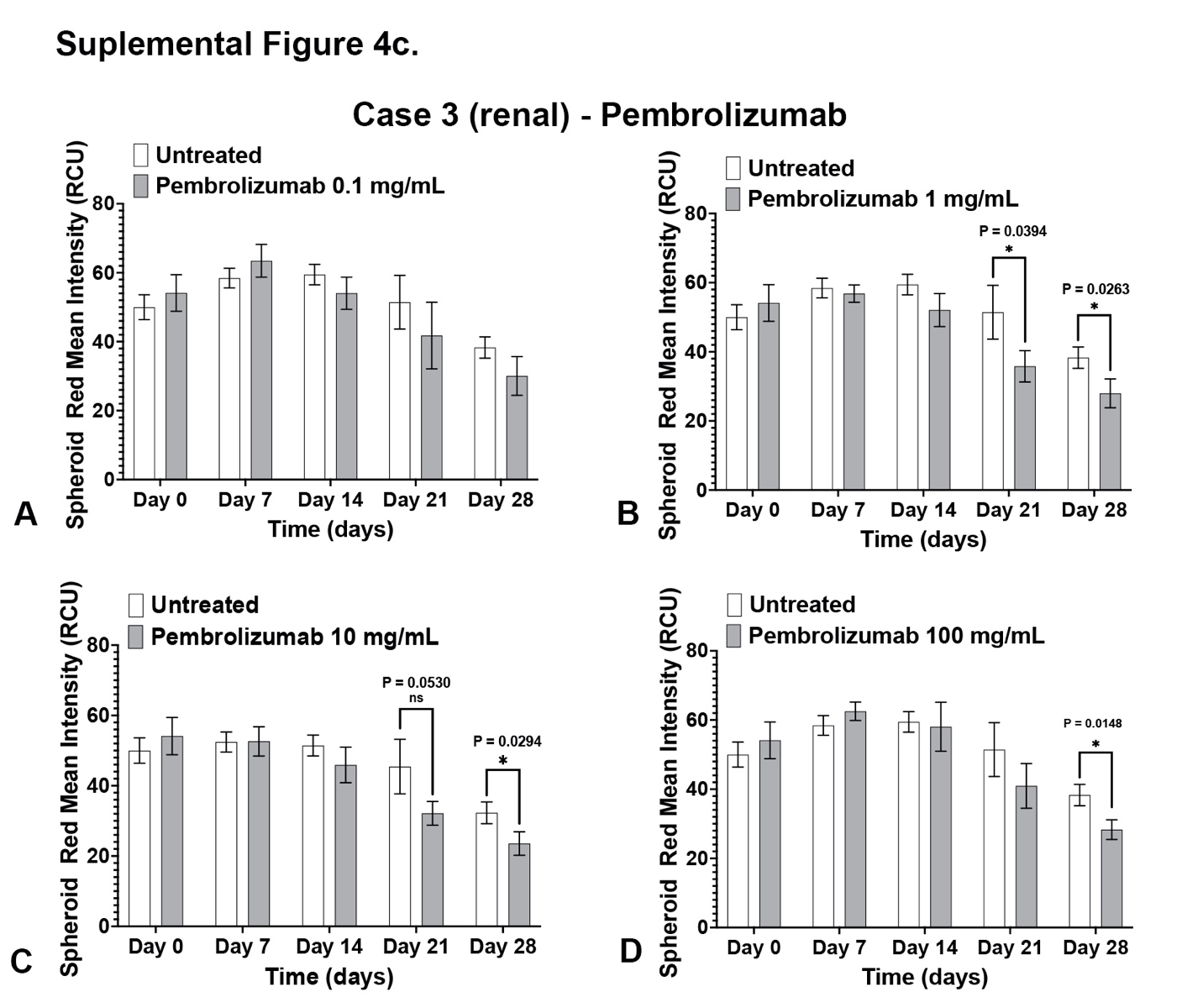


**Supplemental Figure 4c. Clinical response validates the immunotumoroid response to ICIs (Case 3).** Treatment of case 3 with pembrolizumab improved the viability of the immunotumoroids instead of induction of cell death compared to the untreated control. Clinically, the Case 3 patient progressed after 5 doses of nivolumab. Statistical significance was determined by unpaired t-test; Error bars represent the mean ±SD of three independent experiments. *, P ≤ 0.05, "ns" not statistically significant.


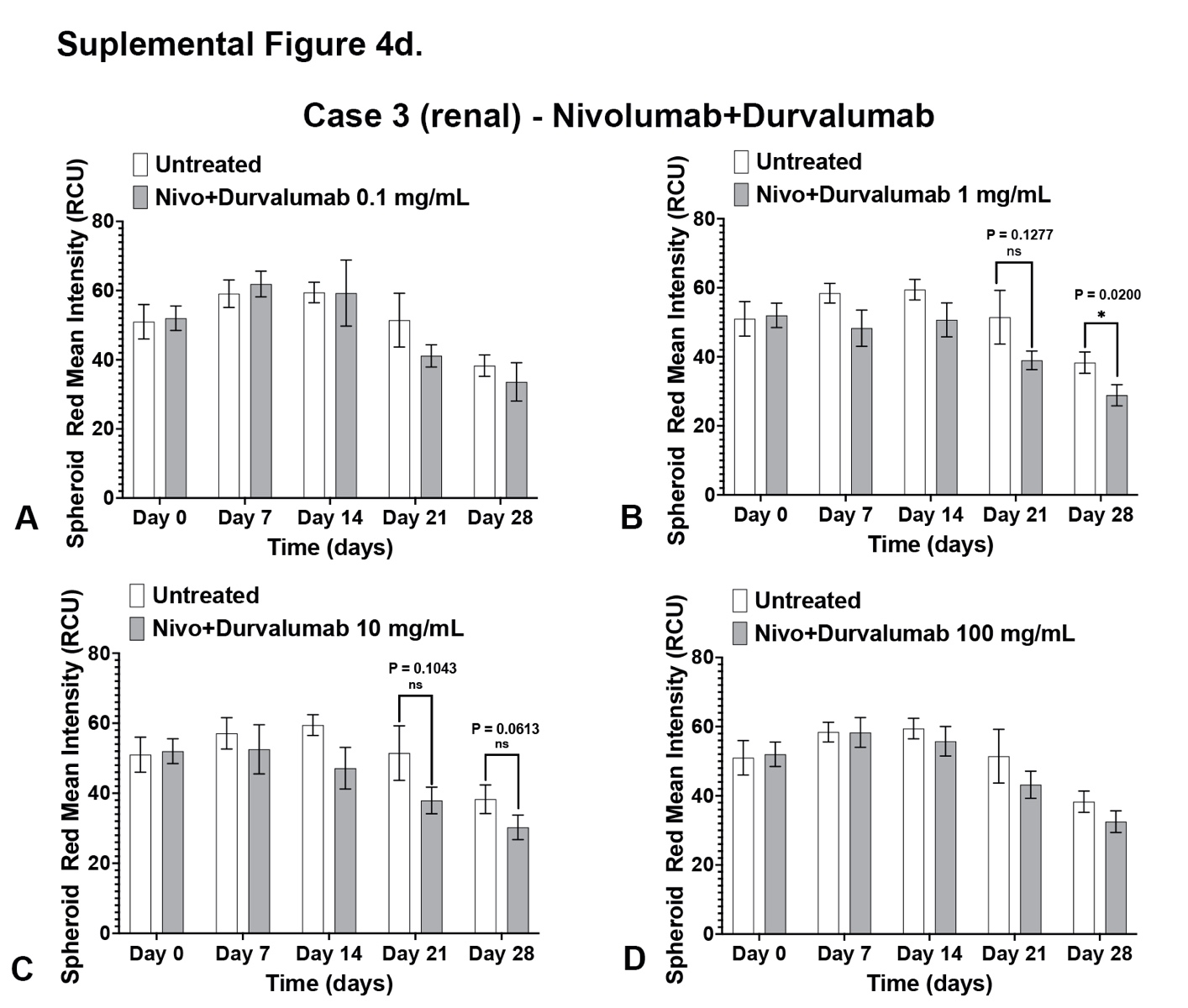


**Supplemental Figure 4d. Clinical response validates the immunotumoroid response to ICIs (Case 3).** Treatment of case 3 with a combination of nivolumab and durvalumab improved the viability of the immunotumoroids instead of induction of cell death compared to the untreated control. Clinically, the Case 3 patient progressed after 5 doses of nivolumab. Statistical significance was determined by unpaired t-test; Error bars represent the mean ±SD of three independent experiments. *, P ≤ 0.05, "ns" not statistically significant.
